# KO*^t^*Bu as a Single Electron Donor? Revisiting the Halogenation of Alkanes with CBr_4_ and CCl_4_

**DOI:** 10.3390/molecules23051055

**Published:** 2018-05-01

**Authors:** Katie J. Emery, Allan Young, J. Norman Arokianathar, Tell Tuttle, John A. Murphy

**Affiliations:** Department of Pure and Applied Chemistry, University of Strathclyde, 295 Cathedral Street, Glasgow G1 1XL, UK; katie.polly.emery@gmail.com (K.J.E.); a.young@strath.ac.uk (A.Y.); jude.arokianathar@strath.ac.uk (J.N.A.)

**Keywords:** halogenation, potassium *tert*-butoxide, alkanes, dihalocarbenes, hypohalite

## Abstract

The search for reactions where KO*^t^*Bu and other *tert*-alkoxides might behave as single electron donors led us to explore their reactions with tetrahalomethanes, CX_4_, in the presence of adamantane. We recently reported the halogenation of adamantane under these conditions. These reactions appeared to mirror the analogous known reaction of NaOH with CBr_4_ under phase-transfer conditions, where initiation features single electron transfer from a hydroxide ion to CBr_4_. We now report evidence from experimental and computational studies that KO*^t^*Bu and other alkoxide reagents do not go through an analogous electron transfer. Rather, the alkoxides form hypohalites upon reacting with CBr_4_ or CCl_4_, and homolytic decomposition of appropriate hypohalites initiates the halogenation of adamantane.

## 1. Introduction

**Scheme 1:**
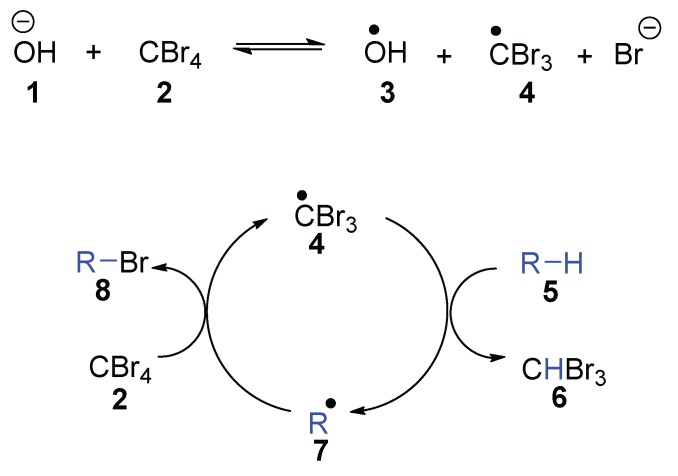
The Schreiner–Fokin mechanism for the bromination of alkanes from reaction of hydroxide with carbon tetrabromide [15].

Recent reports have described transition-metal-free reactions, such as the coupling of haloarenes with arenes [1,2,3,4], that are promoted by a combination of KO*^t^*Bu and an organic additive. The reactions proceed by a radical mechanism, with a single electron transfer (SET) step initiating the formation of the radicals. It has previously been reported that KO*^t^*Bu is capable of reductively activating a range of substrates via single electron transfer [5,6], and this led some authors to propose that *tert*-butoxide anion is responsible for the initiation of these coupling reactions, either alone or in complexation with an additive, such as 1,10-phenanthroline, in the ground state [2,3,7,8,9,10,11]. However, recent publications have shown that the KO*^t^*Bu base reacts with the organic additive to form an electron donor in situ. SET from this electron donor then initiates the radical chain mechanism, not SET from *tert*-butoxide anion [12,13,14]. The investigations begged the question: “under what circumstances would KO*^t^*Bu behave as a single electron donor?” To answer that question, we were drawn to the research of Schreiner and Fokin et al., who reduced CBr_4_ using aqueous NaOH and a phase-transfer catalyst in a two-phase system that led to the bromination of adamantane [15,16]. They demonstrated that reaction of hydroxide anion with CBr_4_ led to the tribromomethyl radical **4**, and concluded that this reflected direct electron transfer from hydroxide to CBr_4_ (Scheme 1). This tribromomethyl radical **4** undergoes a chain reaction, where it abstracts a hydrogen atom from adamantane **5** to form the alkyl radical **7** and bromoform **6**. The adamantyl radical **7** abstracts a bromine atom from CBr_4_, forming adamantyl bromide **8** and regenerating the tribromomethyl radical **4** [15]. The reactions of the tribromomethyl radical show diagnostic high selectivities for abstracting tertiary CH hydrogen atoms (to ultimately form 1-bromoadamantane) over secondary (CH_2_) counterparts (which would ultimately form 2-bromoadamantane).

Recently, we showed that KO*^t^*Bu reacts with CBr_4_ and adamantane (40 °C, 96 h) to afford bromoadamantane—in a reaction that appears to mirror the work of Schreiner and Fokin et al.—with NaOH, although phase-transfer conditions were not used [17]. The oxidation potential of KO*^t^*Bu in DMF (0.10 V vs. SCE) [8] is not too different from the reduction potential of CBr_4_ in DMF (−0.31 V vs. SCE) [18]. It was therefore proposed that KO*^t^*Bu might reduce CBr_4_ through an electron transfer mechanism, but the door was left open to further investigation. The aim of this paper is to explore the chemistry of KO*^t^*Bu and related tertiary alkoxides in this context.

## 2. Results

**Scheme 2:**
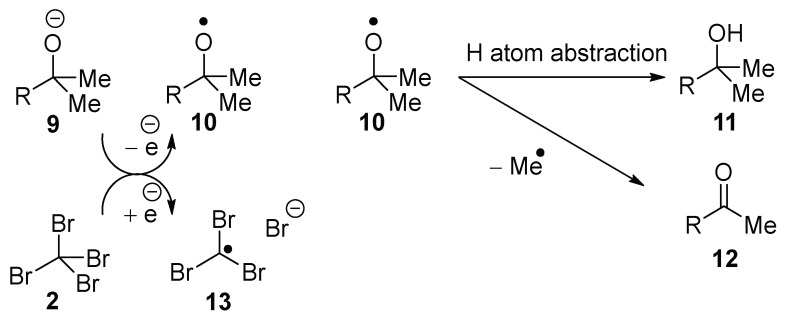
The proposed mechanism for single electron transfer (SET) from *tert*-alkoxides to CBr_4_.

The analogy of the proposed reaction of KO*^t^*Bu with CBr_4_ or CCl_4_ (Scheme 2) to the Schreiner–Fokin reaction of NaOH with this halide is striking (Scheme 1). Upon donation of an electron, the alkoxide anion **9** would form the corresponding alkoxyl radical **10**, which can undergo either hydrogen atom transfer (HAT) to form **11**, or β-scission to form the derived ketone **12** (for appropriate R). Either the alkoxyl radical **10** or the methyl radical can abstract a hydrogen atom from adamantane; the adamantyl radical would then propagate the reaction as in Scheme 1. In order to detect the product from the β-scission of the alkoxyl radical **10**, and bearing in mind the volatility of acetone from β-scission of *tert*-butoxyl radicals, we synthesized potassium 2-phenylpropan-2-olate **14** and used it as a base to explore a range of reaction conditions (Table 1). 

Surprisingly, and in contrast to the case for KO*^t^*Bu, exposure of **14** to CBr_4_ in dichloromethane solvent at 40 °C led to no bromination of adamantane (Table 1, entry 1). The products (2,2-dibromo-1-methylcyclopropyl)benzene **16**, methylstyrene **17** and (2,2-dichloro-1-methylcyclo-propyl)benzene **19** were observed, in addition to 2-phenylpropanol **15** and unreacted adamantane **18** (Table 1, entry 1). To avoid the complexity of having compounds bearing different halogens in the reaction mixture, the reaction was repeated in dichloromethane using CCl_4_ as the reagent, instead of CBr_4_ (Table 1, entry 2). Although we had no evidence of light-sensitivity, we conducted this and all future experiments in foil-covered flasks [19]. The reaction yielded (2,2-dichloro-1-methylcyclopropyl)-benzene **19** as the major product. Note that a blank reaction in dichloromethane (absence of CX_4_) resulted in the formation of product, bis((2-phenylpropan-2-yl)oxy)methane (**20**, 52%) (Table 1, entry 3) [20].

**Scheme 3:**
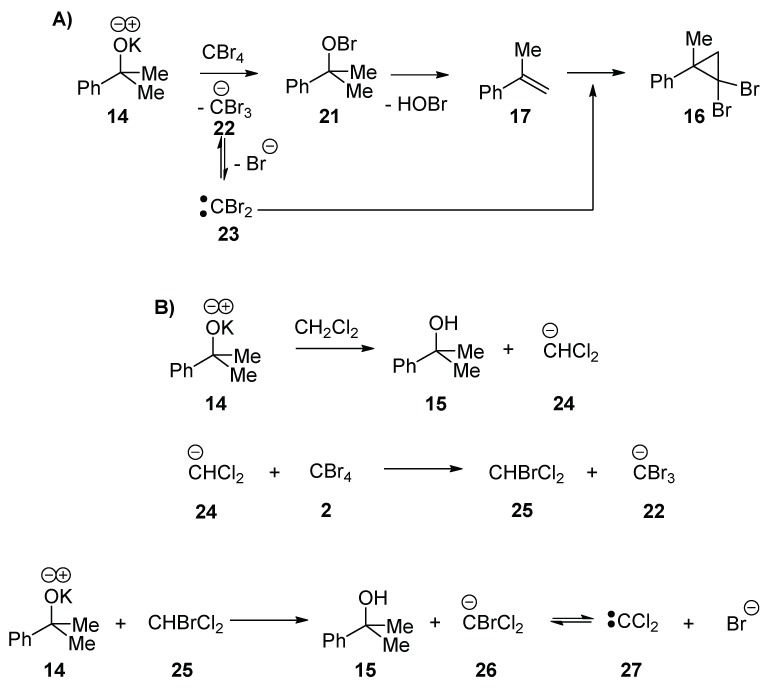
(**A**) The proposed mechanism for the formation of methylstyrene **17** and (2,2-dibromo-1-methylcyclopropyl)-benzene **16** and (**B**) the CCl_2_ carbene **27**, that is used to form (2,2-dichloro-1-methylcyclopropyl)benzene **19** (not shown).

We propose that both (2,2-dibromo-1-methylcyclopropyl)-benzene **16** and (2,2-dichloro-1-methylcyclopropyl)benzene **19** are formed from methylstyrene **17**. The formation of methylstyrene is therefore greatly encouraged in the presence of CBr_4_, consistent with the conversion of the alkoxide to a leaving group (i.e., a hypobromite) (Scheme 3A). It is known that hypobromites are formed from reaction of KO*^t^*Bu with halogens, X_2_ [21] (and that hypohalites are precursors to alkene halogenation by radical mechanisms [21,22]), so this reaction shows that they also form from reaction with tetrahalomethanes. The potassium 2-phenylpropan-2-olate **14** nucleophilically attacks a molecule of CBr_4_ to form the hypobromite **21** and eliminate a CBr_3_ anion **22**. The hypobromite **21** then undergoes an elimination to form methylstyrene **17**. The formation of (2,2-dibromo-1-methylcyclopropyl)benzene **16** occurs by decomposition of the tribromomethyl anion to CBr_2_ **23**, which attacks methylstyrene **17** (Scheme 3A).

Analogous to the formation of (2,2-dibromo-1-methylcyclo-propyl)benzene **16**, the isolation of (2,2-dichloro-1-methylcyclo-propyl)benzene **19** means that carbenes (in this case, CCl_2_ **27**) are formed under the reaction conditions (Scheme 3B). The carbene **27** forms when the potassium 2-phenylpropan-2-olate **14** deprotonates the solvent, CH_2_Cl_2_, and the resulting CHCl_2_ anion **24** undergoes halogen exchange with CBr_4_ to form bromodichloromethane **25**. A second deprotonation would lead to the bromodichloromethyl anion **26**, and decomposition of this anion would lead to the dichlorocarbene **27**. 

**Scheme 4:**
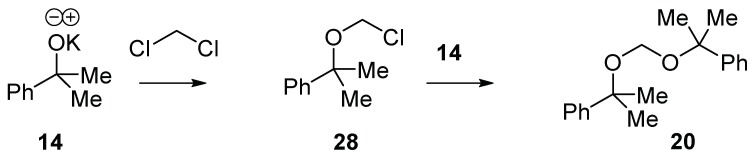
Formation of bis((2-phenylpropan-2-yl)oxy)methane **20**.

In the blank reaction (without CBr_4_) (Table 1, entry 3), bis((2-phenylpropan-2-yl)oxy)methane **20** is formed by the nucleophilic displacement of chloride from dichloromethane by two molecules of potassium 2-phenylpropan-2-olate **14** (Scheme 4) [23,24]. The fact that bis((2-phenylpropan-2-yl)oxy)methane **20** is only observed in the absence of CBr_4_ suggests that the reaction of potassium 2-phenylpropan-2-olate **14** with dichloromethane is slower than the reaction with CBr_4_.

**Scheme 5:**
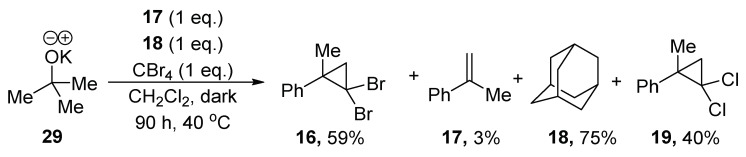
Using methylstyrene **17** to block the bromination of adamantane.

Reflecting on the fact that potassium 2-phenylpropan-2-olate **14** did not give rise to halogenation of adamantane (Table 1), while halogenation was observed when KO*^t^*Bu was used as the alkoxide (the ratio of adamantane **18**:1-bromoadamantane **31**:2-bromoadamantane **51** was determined to be 39:3.3:1 [17]), this suggests that either the two alkoxide bases do not react with CBr_4_ through analogous mechanisms, or that the halogenation reaction was intercepted and inhibited when **14** was used. Interception could occur if methylstyrene **17**—formed in the reaction from potassium 2-phenylpropan-2-olate **14**—shuts down the radical chain pathway. To probe this possibility, the reaction of KO*^t^*Bu with CBr_4_ and adamantane **18** was repeated in the presence of methylstyrene **17** (1 equiv., Scheme 5).

The addition of methylstyrene **17** completely inhibited the bromination of adamantane, which suggests that it can shut down the radical mechanism, perhaps by acting as a preferential source of hydrogen atoms, relative to adamantane **18**, for the radical intermediates in the reaction. Computationally, the competition between methylstyrene and adamantane as a source of H atoms was modeled using CBr_3_ radicals, which showed that hydrogen atom abstraction by the CBr_3_ radical from methylstyrene **17** (ΔG^ǂ^ = 10.7 kcal/mol and ΔG_rxn_ = −7.7 kcal/mol) is thermodynamically and kinetically more favorable than from adamantane **18** (ΔG^ǂ^ = 13.9 kcal/mol and ΔG_rxn_ = 1.1 kcal/mol) (see Supplementary Materials).

All yields were calculated using 1,3,5-trimethoxybenzene as the internal standard (10 mol %) in the ^1^H-NMR. The yield of **18** was calculated based on recovery of adamantane **18**, the yield of **16** and **19** was calculated based on CBr_4_. (Note that alkoxide (4 eq.) is capable of forming methylstyrene **17**.)

With the knowledge that methylstyrene **17** is capable of preventing bromination of adamantane, why does bromination succeed when KO*^t^*Bu **29** + CBr_4_ alone are used? The two hypobromites, *tert*-butyl hypobromite and **21**, may undergo elimination at different rates; additionally, 2-methylpropene (b.p. −6.9 °C) exists as a gas at the reaction temperature. Such a volatile product would likely be found principally in the headspace above the reaction, rather than in solution, and therefore halogenation of adamantane **18** could occur.

As mentioned earlier, Wirth et al. [19] used *tert*-butyl hypobromite to achieve the bromination of alkanes (while Walling [20] used *tert*-butyl hypochlorite to achieve chlorination) and proposed that the mechanism was initiated via homolysis of the O–Br bond of *tert*-butyl hypobromite. Either the bromine radical or the *tert*-butoxyl radical could abstract the H atom from adamantane. Propagation could then occur when the adamantyl radical abstracts a Br atom from CBr_4_ [13] or from *tert*-butyl hypobromite [23]. 

To gain further information on mechanism, an alternative alkoxide, potassium triphenylmethanolate **30**, was prepared and subjected to the reaction conditions with CBr_4_ and adamantane **18** (Table 2).

Reaction of potassium triphenylmethanolate **30** with adamantane in the presence of CBr_4_ afforded products 1-bromoadamantane **31** (7%) and triphenylmethanol **32** (88%), as well as two additional products, benzophenone **33** (5%) and 4-benzhydrylphenol **34** (12%) (Table 2, entry 1). When CBr_4_ was not present in the reaction mixture, triphenylmethanol **32** (81%) was isolated following workup, together with a trace (1%) of benzophenone **33** (Table 2, entry 2). Further analysis of the starting material showed trace amounts of **33** present in the commercially supplied triphenylmethanol **32**. Background formation of benzophenone **33**, in trace amounts from heterolytic fragmentation of similar tertiary alkoxides with expulsion of a phenyl anion, is also precedented [25,26]. 

**Scheme 6:**
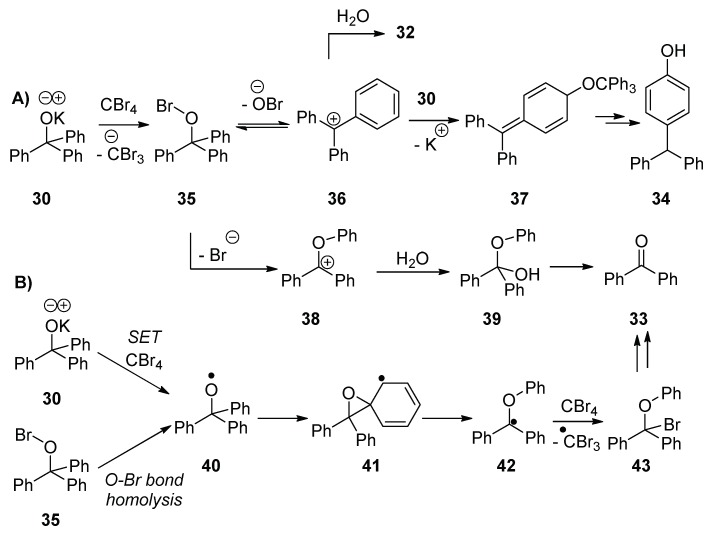
Proposed pathway for the formation of **32**, **33** and **34** from **30**: (**A**) through ionic intermediates and (**B**) through radical intermediates.

The important difference between the reaction in the presence of CBr_4_ and in its absence (Table 2, entry 1 and entry 2, respectively) is the formation of 4-benzhydrylphenol **34**, which can arise through the hypobromite **35** (Scheme 6). Potassium triphenylmethanolate **30** reacts with CBr_4_ to generate the hypobromite **35**. This hypobromite can react by three pathways (1) the OBr anion leaves to form the stabilized cation **36**; (2) the O–Br bond fragments ionically with simultaneous migration of a phenyl moiety to form cation **38**; or (3) the O–Br bond undergoes homolysis to form alkoxyl radical **40** and a bromine radical, for which there is literature precedent [23]. If pathway (1) is followed, the carbocation **36** is attacked by another molecule of potassium triphenylmethanolate **30** and, due to steric effects, the alkoxide attacks at the *para* position of one of the benzene rings. In doing so, the species **37** is formed, which, following tautomerism and hydrolytic workup, leads to **34**. Alternatively, **36** affords triphenylmethanol **32** on workup. Pathways (2) and (3) ultimately lead to the formation of benzophenone **33**; pathway (2) involves the formation of intermediate **38**, which reacts with water on workup to form **39** and, ultimately, benzophenone **33**. Pathway (3) involves formation of the alkoxyl radical **40**, which could form via O–Br homolysis of **35** or, alternatively, by SET from potassium triphenylmethanolate **30** to a molecule of CBr_4_. This alkoxyl radical might undergo β-scission to form benzophenone **33**. However, alternatively, the radical **40** could undergo a neophyl-like rearrangement to form radical **41** (Scheme 6) [27]. The product **43** forms when the radical **42** abstracts a bromine atom from either CBr_4_ or the hypobromite **35**, and **43**, in turn, upon workup, undergoes a hydrolytic conversion to benzophenone **33**. Previous fragmentations of related alkoxyl radicals have been studied [28,29,30,31,32]. The enhanced formation of benzophenone **33** in the presence of CBr_4_ could occur through either β-scission of the alkoxyl radical **40** (Scheme 6B), or the ionic mechanism (Scheme 6A).

The study above provides evidence for significant formation of hypohalites from alkoxides **14** and **30**. However, it might be possible for the O*^t^*Bu anion in KO*^t^*Bu to behave differently. The absence of electron-withdrawing aryl groups would render it more electron-rich, and, therefore, it might be a better candidate than the other alkoxides to undergo electron transfer.

**Figure 1:**
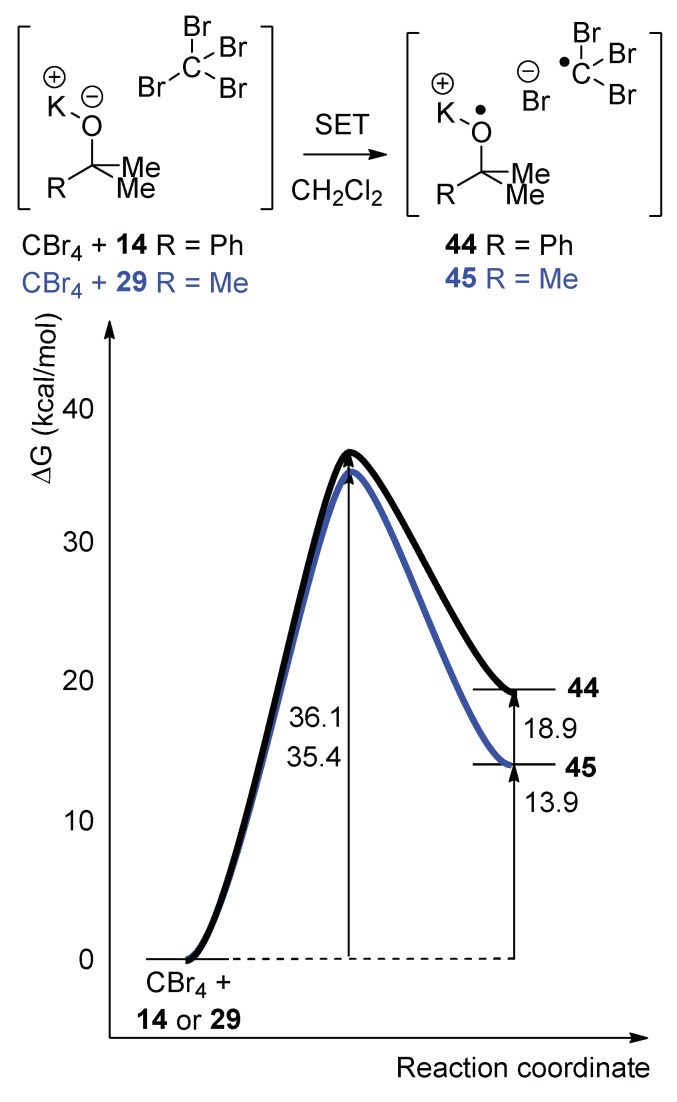
Computational modelling of SET from alkoxides, KO*^t^*Bu and potassium 2-phenylpropan-2-olate **14** to CBr_4_ in dichloromethane.

To probe the ability of alkoxides to donate a single electron, computational modeling was implemented to calculate the energy profile for the SET from both KO*^t^*Bu and potassium 2-phenylpropan-2-olate **14** to CBr_4_ in dichloromethane (Figure 1) [33]. Our previous calculations had been based on the classical Nelsen four-point method [34], but, since that time, we published a more accurate complexation method [33] for predicting the activation free energy and the relative free energy of reactions. (The calculations were conducted using the M06-2X functional [35,36] with the 6-311++G(d,p) basis set [37,38,39,40,41] on all atoms, except for the bromine. Bromine was modeled with the MWB28 relativistic pseudo-potential and associated basis set [42]. All calculations were carried out using the C-PCM implicit solvent model [43,44] with the dielectric constant for dichloromethane (ε = 8.93) or carbon tetrachloride (ε = 2.228) as appropriate. All calculations were performed in Gaussian09 [45]). 

The energy barriers for SET from either KO*^t^*Bu or potassium 2-phenylpropan-2-olate **14**, to a molecule of CBr_4_ were calculated to be ΔG^ǂ^ = +35.4 kcal/mol and +36.1 kcal/mol, respectively (while the corresponding ΔG_rxn_ values were +13.9 and +18.9 kcal/mol) (Figure 1). When the energy barrier was calculated for the reactions with CCl_4_, a similar energy profile was obtained for the SET from either of the two alkoxides to a molecule of CCl_4_ (see Supplementary Materials). The energy barriers calculated were ΔG^ǂ^ = 42.5 kcal/mol and 44.5 kcal/mol for SET to CCl_4_ from KO*^t^*Bu and potassium 2-phenylpropan-2-olate **14**, respectively. These barriers for reactions of both CBr4 and CCl_4_ are not accessible for a reaction performed at 40 °C, even as an initiation step. These computationally derived energy profiles for the SET step indicate that the initiation for this halogenation of adamantane **18** is not via SET from the alkoxide; the likely alternative radical pathway arises through homolysis of the hypohalite intermediate [19]. (Importantly, our computation predicts a barrierless, but endergonic, profile for homolysis of the O–Br bond in *^t^*BuO–Br with ΔG_rxn_ = +27.6 kcal/mol [34]. This is a highly endergonic reaction, but as it is simply an initiation step, few *^t^*BuO–Br molecules are required to undergo homolysis).

## 3. Experimental Section

### 3.1. Computational Methods

The calculations were run using the M06-2X functional [35,36] with the 6-311++G(d,p) basis set [37,38,39,40,41] on all atoms except bromine. Bromine was modeled with the MWB28 relativistic pseudo-potential and associated basis set [42]. All calculations were carried out using the C-PCM implicit solvent model [43,44] as implemented in Gaussian09 [45].

### 3.2. General Experimental Information

All reagents were bought from commercial suppliers and used without further purification unless stated otherwise. All the reactions were carried out under argon atmosphere. Diethyl ether, tetrahydrofuran, dichloromethane and hexane were dried with a Pure-Solv 400 solvent purification system, marketed by Innovative Technology Inc. (Herndon, VA, USA). Organic extracts were, in general, dried over anhydrous sodium sulfate (Na_2_SO_4_). A Büchi rotary evaporator was used to concentrate the reaction mixtures. Thin layer chromatography (TLC) was performed using aluminium-backed sheets of silica gel and visualized under a UV lamp (254 nm). The plates were developed using phosphomolybdic acid or KMnO_4_ solution. Column chromatography was performed to purify compounds by using silica gel 60 (200–400 mesh). 

The electron transfer reactions were carried out within a glove box (Innovative Technology Inc.) under nitrogen atmosphere, and performed in an oven-dried or flame-dried apparatus using anhydrous solvents, which were degassed under reduced pressure, then purged with argon and dried over activated molecular sieves (3 Å), prior to being sealed and transferred to the glovebox. All solvent or samples placed into the glovebox were transferred through the port, which was evacuated and purged with nitrogen 10 times before entry. When the reaction mixtures were prepared, the reaction vessel was removed from the glove box and the rest of the reaction was performed in a fumehood.

Proton (^1^H) NMR spectra were recorded at 400.13, 400.03 and 500.16 MHz on Bruker AV3, AV400 and AV500 spectrometers, respectively. Carbon (^13^C) NMR spectra were recorded using broadband decoupled mode at 100.61, 100.59 and 125.75 MHz on Bruker AV3, AV400 and AV500 spectrometers, respectively. Spectra were recorded in either deuterated chloroform (CDCl_3_) or deuterated dimethyl sulfoxide (d^6^-DMSO), depending on the solubility of the compounds. The chemical shifts are reported in parts per million (ppm), calibrated on the residual non-deuterated solvent signal, and the coupling constants, *J*, are reported in Hertz (Hz). The peak multiplicities are denoted using the following abbreviations: s, singlet; d, doublet; t, triplet; q, quartet; sx, sextet; m, multiplet; br s, broad singlet; dd, doublet of doublets; dt, doublet of triplets; td, triplet of doublets.

Infrared spectra were recorded on an ATR-IR spectrometer. Melting points were determined on a Gallenkamp melting point apparatus. The mass spectra were recorded by either gas-phase chromatography (GCMS) or liquid-phase chromatography (LCMS), using various ionization techniques, as stated for each compound: atmospheric pressure chemical ionization (APCI), electron ionization (EI), electrospray ionization (ESI). GCMS data were recorded using an Agilent Technologies 7890A GC system coupled to a 5975C inert XL EI/CI MSD detector. Separation was performed using the DB5MS-UI column (30 m × 0.25 mm × 0.25 μm) at a temperature of 320 °C, using helium as the carrier gas. LCMS data were recorded using an Agilent 6130 Dual source mass spectrometer with Agilent 1200, Agilent Poroshell 120ECC 4.6 mm × 75 mm × 2.7 um column. 

High-resolution mass spectrometry (HRMS) was performed at the University of Wales, Swansea, in the EPSRC National Mass Spectrometry Centre. Accurate mass was obtained using atmospheric pressure chemical ionization (APCI), chemical ionization (CI), electron ionization (EI), electrospray ionization (ESI) or nanospray ionization (NSI) with a LTQ Orbitrap XL mass spectrometer.

### 3.3. Synthesis of Alkoxide, Potassium 2-Phenylpropan-2-Olate ***14***

Potassium hydride (586 mg, 15 mmol, 1.0 eq.) was added to a flame-dried three-necked flask, equipped with a vacuum tap. Under an argon atmosphere, at −78 °C, a solution of 2-phenylpropanol **15** (2.04 g, 15 mmol) in anhydrous diethyl ether (20 mL), as added and the reaction mixture, was stirred at −78 °C for 1 h, then at RT overnight. The solvent was removed under vacuum and the crude material was dried for 1 h to obtain potassium 2-phenylpropan-2-olate **14** (2.46 g, 14.1 mmol, 93%) as an off-white solid m.p. 128–132 °C; (Found: (GCMS-EI) C_9_H_11_O (M-K) 135.08); ν_max_ (film)/cm^−1^ 3503, 2972, 1663, 1444, 1433, 1236, 1161, 1067, 1029, 955, 881, 861, 764; ^1^H-NMR (400 MHz, d^6^-DMSO) δ 1.41 (6 H, s, 2 × CH_3_), 7.15–7.19 (1 H, m, ArH), 7.26–7.30 (2 H, m, ArH), 7.45–7.47 (2 H, m, ArH); ^13^C-NMR (100 MHz, *d*_6_-DMSO) δ 31.9 (2 × CH_3_), 70.5 (C), 124.4 (2 × CH), 125.8 (CH), 127.7 (2 × CH), 150.5 (C). The product was put under an argon atmosphere and transported into the glovebox immediately.

### 3.4. Blank Reaction (No KO^t^Bu) of CBr_4_ with Adamantane ***18***

Adamantane **18** (68 mg, 0.5 mmol), CBr_4_ (166 mg, 0.5 mmol, 1.0 eq.) and dichloromethane (3.13 mL) were added to an oven-dried pressure tube and the reaction mixture was stirred at 40 °C for 90 h in the dark. The reaction mixture was cooled to RT and quenched with aqueous hydrochloric acid (1 M, 5 mL) and extracted with diethyl ether (4 × 10 mL). The organic phases were combined, dried over Na_2_SO_4_, filtered and concentrated in vacuo. ^1^H-NMR (400 MHz, CDCl_3_) δ 1.76–1.75 (12 H, m, C*H*_2_), 1.88 (4 H, br s, C*H*); ^13^C-NMR (100 MHz, CDCl_3_) δ 28.5 (6 × CH_2_), 37.9 (4 × CH). (The yield of adamantane **18** [46] (93%) was determined by adding 1,3,5-trimethoxybenzene to the crude mixture as an internal standard for ^1^H-NMR). These signals are consistent with the literature values and reference samples.

### 3.5. Reactions of Potassium 2-Phenylpropan-2-Olate ***14*** with Adamantane ***18*** and CBr_4_/CCl_4_

#### 3.5.1. Table 1, Entry 1

Potassium 2-phenylpropan-2-olate **14** (349 mg, 2 mmol, 4.0 eq.), adamantane **18** (68 mg, 0.5 mmol), CBr_4_ (166 mg, 0.5 mmol, 1.0 eq.) and dichloromethane (3.13 mL) were added to an oven-dried pressure tube, and the reaction mixture was stirred at 40 °C for 90 h. The reaction mixture was cooled to RT and quenched with aqueous hydrochloric acid (1 M, 5 mL) and extracted with diethyl ether (4 × 10 mL). The organic phases were combined, dried over Na_2_SO_4_, filtered and concentrated in vacuo. The yield of 2-phenylpropanol **15** (66%), (2,2-dibromo-1-methylcyclopropyl)-benzene **16** (33%), methylstyrene **17** (18%), adamantane **18** (91%) and (2,2-dichloro-1-methylcyclopropyl)benzene **19** (17%) were determined by adding 1,3,5-trimethoxybenzene to the crude mixture as an internal standard for ^1^H-NMR. The products were identified by the following characteristic signals; ^1^H-NMR (400 MHz, CDCl_3_) δ 1.60 (6 H, s) for 2-phenylpropanol **15**; δ 1.72 (3 H, s), 1.78 (1 H, d, *J* = 7.6 Hz), 2.17 (1 H, d, *J* = 7.6 Hz) for (2,2-dibromo-1-methylcyclopropyl)benzene **16**; δ 2.16 (3 H, s), 5.09 (1 H, s), 5.37 (1 H, s) for methylstyrene **17**; δ 1.75–1.77 (12 H, m), 1.88 (4 H, br s) for adamantane **18**; δ 1.68 (3 H, s), 1.96 (1 H, d, *J* = 7.2 Hz) for (2,2-dichloro-1-methylcyclopropyl)benzene **19**; ^13^C-NMR (100 MHz, CDCl_3_) δ 31.9, 72.7, 124.5, 126.8, 128.4 for 2-phenylpropanol **15**; δ 27.9, 33.9, 128.5, 128.6 for (2,2-dibromo-1-methylcyclopropyl)-benzene **16**; δ 22.0, 112.5 for methylstyrene **17**; δ 28.5, 37.9 for adamantane **18**; δ 25.7, 36.6 for (2,2-dichloro-1-methylcyclo-propyl)benzene **19**. These signals are consistent with the literature values and reference samples. The compounds **16** and **19** were inseparable, so pure samples of **16** and **19** were prepared for comparison- see below.

#### 3.5.2. Preparation of **16**

KO*^t^*Bu **29** (224 mg, 2 mmol, 4.0 eq.), HCBr_3_ (0.04 mL, 0.5 mmol, 1.0 eq.) and methylstyrene **17** (0.07 mL, 0.5 mmol) were added to an oven-dried pressure tube. Dichloromethane (3.13 mL) was added and the reaction mixture was stirred at 40 °C for 90 h. The reaction mixture was cooled to RT and quenched with aqueous hydrochloric acid (1 M, 5 mL) and extracted with diethyl ether (4 × 10 mL). The organic phases were combined, dried over Na_2_SO_4_, filtered and concentrated in vacuo. The crude material was purified by column chromatography (100% hexane) to give (2,2-dibromo-1-methylcyclopropyl)benzene **16** [18] (82.4 mg, 57%) as a colorless oil [Found: (GCMS-CI) C_10_H_11_Br_2_^+^ (M + H)^+^ 288.7]; ν_max_ (film)/cm^−1^ 1496, 1445, 1426, 1060, 1019, 763, 691; ^1^H-NMR (400 MHz, CDCl_3_) δ 1.72 (3 H, s, CH_3_), 1.78 (1 H, d, *J* = 7.6 Hz, CH_2_), 2.17 (1 H, d, *J* = 7.6 Hz, CH_2_), 7.29–7.38 (5 H, m, ArH); ^13^C{^1^H}-NMR (100 MHz, CDCl_3_) δ 27.9 (CH_3_), 33.8 (CH_2_), 35.9 (C), 36.9 (C), 127.4 (CH), 128.5 (2 × CH), 128.6 (2 × CH), 142.5 (C); m/z (CI) 292.6 [(M + H)^+^, ^81^Br^81^Br, 61%), 290.6 [(M + H)^+^, ^79^Br^81^Br, 100), 288.7 [(M + H)^+^, ^79^Br^79^Br, 70)]. 

#### 3.5.3. Preparation of **19**

KO*^t^*Bu **29** (224 mg, 2 mmol, 4.0 eq.), HCCl_3_ (0.04 mL, 0.5 mmol, 1.0 eq.) and methylstyrene **17** (0.07 mL, 0.5 mmol) were added to an oven-dried pressure tube. Dichloromethane (3.13 mL) was added and the reaction mixture was stirred at 40 °C for 90 h. The reaction mixture was cooled to RT and quenched with aqueous hydrochloric acid (1 M, 5 mL) and extracted with diethyl ether (4 × 10 mL). The organic phases were combined, dried over Na_2_SO_4_, filtered and concentrated in vacuo. The crude material was purified by column chromatography (100% hexane) to give (2,2-dichloro-1-methylcyclopropyl)benzene **19** [18] (63.7 mg, 63%) as a colorless oil [Found: (HRMS-EI) 200.0157. C_10_H_10_Cl_2_ (M)^•+^ requires 200.0160]; ν_max_ (film)/cm^−1^ 1497, 1446, 1425, 1075, 1033, 1026, 936, 868, 772, 754, 697, 595; ^1^H-NMR (400 MHz, CDCl_3_) δ 1.60 (1 H, d, *J* = 7.2 Hz, CH_2_), 1.68 (3 H, s, CH_3_), 1.96 (1 H, d, *J* = 7.2 Hz, CH_2_), 7.27–7.38 (5 H, m, ArH); ^13^C-NMR (100 MHz, CDCl_3_) δ 25.7 (CH_3_), 32.0 (CH_2_), 36.6 (C), 66.0 (C), 127.4 (CH), 128.6 (2 × CH), 128.7 (2 × CH), 141.4 (C); m/z (CI) 203.9 ((M)^•+^, ^37^Cl^37^Cl, 12%), 201.9 ((M)^•+^, ^35^Cl^37^Cl, 70), 199.9 ((M)^•+^, ^35^Cl^35^Cl, 100).

#### 3.5.4. Table 1, Entry 2

Potassium 2-phenylpropan-2-olate **14** (349 mg, 2 mmol, 4.0 eq.), adamantane **18** (68 mg, 0.5 mmol), CCl_4_ (0.05 mL, 0.5 mmol, 1.0 eq.) and dichloromethane (3.13 mL) were added to an oven-dried pressure tube and the reaction mixture was stirred at 40 °C for 90 h in the dark. The reaction mixture was cooled to RT and quenched with aqueous hydrochloric acid (1 M, 5 mL) and extracted with diethyl ether (4 × 10 mL). The organic phases were combined, dried over Na_2_SO_4_, filtered and concentrated in vacuo. The yield of 2-phenylpropanol **15** (67%), methylstyrene **17** (3%), adamantane **18** (76%), (2,2-dichloro-1-methylcyclopropyl)benzene **19** (50%) and bis((2-phenylpropan-2-yl)oxy)-methane **20** (3%) were determined by adding 1,3,5-trimethoxybenzene to the crude mixture as an internal standard for ^1^H-NMR. The products were identified by the following characteristic signals; ^1^H-NMR (400 MHz, CDCl_3_) δ 1.60 (6 H, s) for 2-phenylpropanol **15**; δ 2.17 (3 H, s), 5.10 (1 H, s), 5.38 (1 H, s) for methylstyrene **17**; δ 1.76–1.78 (12 H, m), 1.89 (4 H, br s) for adamantane **18**; δ 1.68 (3 H, s), 1.97 (1 H, d, *J* = 7.2 Hz) for (2,2-dichloro-1-methylcyclopropyl)benzene **19**; δ 4.51 (2 H, s), 7.20–7.24 (2 H, m) for bis((2-phenylpropan-2-yl)oxy)methane **20**; ^13^C-NMR (100 MHz, CDCl_3_) δ 31.9, 72.7, 124.5, 126.8, 128.4 for 2-phenylpropanol **15**; δ 22.0, 112.6 for methylstyrene **17**; δ 28.5, 37.9 for adamantane **18**; δ 25.6, 36.6 for (2,2-dichloro-1-methylcyclopropyl)benzene **19**. These signals are consistent with the literature values and reference samples.

#### 3.5.5. Table 1, Entry 3 

Potassium 2-phenylpropan-2-olate **14** (349 mg, 2 mmol, 4.0 eq.), adamantane **18** (68 mg, 0.5 mmol) and dichloromethane (3.13 mL) were added to an oven-dried pressure tube and the reaction mixture was stirred at 40 °C for 90 h in the dark. The reaction mixture was cooled to RT and quenched with aqueous hydrochloric acid (1 M, 5 mL) and extracted with diethyl ether (4 × 10 mL). The organic phases were combined, dried over Na_2_SO_4_, filtered and concentrated in vacuo. The yield of 2-phenylpropanol **15** (39%), methylstyrene **17** (1%), adamantane **18** (84%) and bis((2-phenylpropan-2-yl)oxy)methane **20** (52%) were determined by adding 1,3,5-trimethoxybenzene to the crude mixture as an internal standard for ^1^H-NMR. The products were identified by the following characteristic signals; ^1^H-NMR (400 MHz, CDCl_3_) δ 1.60 (6 H, s) for 2-phenylpropanol **15**; δ 2.16 (3 H, s), 5.09 (1 H, s), 5.37 (1 H, s) for methylstyrene **17**; δ 1.75–1.77 (12 H, m), 1.88 (4 H, br s) for adamantane **18**; δ 4.51 (2 H, s), 7.20–7.24 (2 H, m) for bis((2-phenylpropan-2-yl)oxy)methane **20**; ^13^C-NMR (100 MHz, CDCl_3_) δ 31.9, 72.7, 124.5, 149.2 for 2-phenyl-2-propanol **15**; δ 28.5, 37.9 for adamantane **18**; δ 29.5, 77.7, 86.8, 146.9 for bis((2-phenylpropan-2-yl)oxy)methane **20**. These signals are consistent with the literature values and reference samples. This crude material was purified by column chromatography (0–5% ethyl acetate in hexane) to give bis((2-phenylpropan-2-yl)oxy)methane **20** (44.7 mg, 26%) as a colorless oil (Found: (HRMS-ESI) 302.2118. C_19_H_28_O_2_N (M + NH_4_)^+^ requires 302.2115); *ν*_max_ (film)/cm^−1^ 2978, 2934, 1493, 1447, 1381, 1364, 1258, 1153, 1072, 1018, 991, 818, 762; ^1^H-NMR (400 MHz, CDCl_3_) δ 1.59 (12 H, s, 4 × CH_3_), 4.50 (2 H, s, CH_2_), 7.20–7.23 (2 H, m, ArH), 7.27–7.31 (4 H, m, ArH), 7.38–7.40 (4 H, m, ArH); ^13^C-NMR (100 MHz, CDCl_3_) δ 29.5 (4 × CH_3_), 77.7 (2 × C), 86.8 (CH_2_), 125.9 (4 × CH), 126.9 (2 × CH), 128.2 (4 × CH), 146.9 (2 × C).

### 3.6. Reaction of KO^t^Bu in Dichloromethane 

KO*^t^*Bu **29** (224 mg, 2 mmol, 4.0 eq.), CBr_4_ (166 mg, 0.5 mmol, 1.0 eq.), adamantane **18** (68 mg, 0.5 mmol) and dichloromethane (3.13 mL) were added to an oven-dried pressure tube and the reaction mixture was stirred at 40 °C for 90 h in the dark. The reaction mixture was cooled to RT and quenched with aqueous hydrochloric acid (1 M, 5 mL) and extracted with diethyl ether (4 × 10 mL). The organic phases were combined, dried over Na_2_SO_4_, filtered and concentrated in vacuo. The ratio of adamantane **18**:1-bromoadamantane **31**:2-bromoadamant-ane **51** was determined to be 39:3.3:1 from the ^1^H-NMR spectrum of the crude mixture. The products were identified by the following characteristic signals; ^1^H-NMR (400 MHz, CDCl_3_) δ 1.74–1.76 (12 H, m, CH_2_ × 6), 1.88 (4 H, br s, CH × 4) for adamantane **18**; δ 1.73 (6 H, m, CH_2_ × 3), 2.10 (3 H, br s, CH × 3), 2.36 (6 H, m, CH_2_ × 3) for 1-bromoadamantane **31** [47]; δ 1.96–2.00 (2 H, m, CH_2_), 2.15 (2 H, br s, CH × 2), 2.33 (2 H, br s, CH × 2), 4.68 (1 H, br s, C*H*) for 2-bromoadamantane **51** [48]; ^13^C-NMR (100 MHz, CDCl_3_) δ 28.5, 37.9 adamantane **18**; δ 32.8, 35.7, 49.5 for 1-bromoadamantane **31**; 36.6, 39.1 for 2-bromoadamantane **51**. These signals are consistent with the literature values and reference samples.

### 3.7. Reactions of Adamantane ***18*** with CBr_4_, Methylstyrene ***17*** and KO^t^Bu ***29*** (Scheme 5)

KO*^t^*Bu **29** (224 mg, 2 mmol, 4.0 eq.), CBr_4_ (166 mg, 0.5 mmol, 1.0 eq.), methylstyrene **17** (0.07 mL, 0.5 mmol, 1.0 eq.), adamantane **18** (68 mg, 0.5 mmol) and dichloromethane (3.13 mL) were added to an oven-dried pressure tube and the reaction mixture was stirred at 40 °C for 90 h in the dark. The reaction mixture was cooled to RT and quenched with aqueous hydrochloric acid (1 M, 5 mL) and extracted with diethyl ether (4 × 10 mL). The organic phases were combined, dried over Na_2_SO_4_, filtered and concentrated in vacuo. The yield of (2,2-dibromo-1-methylcyclopropyl)benzene **16** (59%), methylstyrene **17** (3%), adamantane **18** (75%) and (2,2-dichloro-1-methylcyclopropyl)benzene **19** (40%) were determined by adding 1,3,5-trimethoxybenzene to the crude mixture as an internal standard for ^1^H-NMR. The products were identified by the following characteristic signals; ^1^H-NMR (400 MHz, CDCl_3_) δ 1.72 (3 H, s), 1.78 (1 H, d, *J* = 7.6 Hz) for (2,2-dibromo-1-methylcyclopropyl)benzene **16**; δ 5.09 (1 H, s), 5.37 (1 H, s) for methylstyrene **17**; δ 1.75–1.78 (12 H, m), 1.88 (4 H, br s) for adamantane **18**; δ 1.60 (1 H, d, *J* = 7.2 Hz), 1.69 (3 H, s), 1.97 (1 H, d, *J* = 7.2 Hz) for (2,2-dichloro-1-methylcyclopropyl)benzene **19**; ^13^C-NMR (100 MHz, CDCl_3_) δ 27.9, 33.9, 36.9, 142.5 for (2,2-dibromo-1-methylcyclopropyl)benzene **16**; δ 28.5, 37.9 for adamantane **18**; δ 25.7, 32.0, 36.6, 141.4 for (2,2-dichloro-1-methylcyclopropyl)benzene **19**. These signals are consistent with the literature values and reference samples [49,50].

### 3.8. Synthesis of Alkoxide, Potassium Triphenylmethanolate ***30***

Potassium hydride (802 mg, 20 mmol, 1.0 eq.) was added to a flame-dried three-necked flask, equipped with a vacuum tap. Under an argon atmosphere, at –78 °C, a solution of triphenylmethanol **32** (5.21 g, 20 mmol) in anhydrous tetrahydrofuran (25 mL) was added and the reaction mixture was stirred at −78 °C for 1 h, then at RT overnight. The solvent was removed on the house vacuum line and the crude material was dried for 1 h to give potassium triphenylmethanolate **30** (5.07 g, 17 mmol, 85%) as an off-white solid, m.p. 238 °C (dec.); (Found: (GCMS-EI) C_19_H_16_O (M)^•+^ 260.1 (under the MS analysis **30** is protonated to the alcohol)); *ν*_max_ (film)/cm^−1^ 3057, 3022, 1595, 1487, 1443, 1414, 1329, 1155, 1053, 1009, 891, 756; ^1^H-NMR (400 MHz, d^6^-DMSO) δ 6.93–6.98 (3 H, m, ArH), 7.04–7.08 (6 H, m, ArH), 7.34–7.37 (6 H, m, ArH); ^13^C-NMR (100 MHz, d^6^-DMSO) δ 84.7 (C), 123.6 (3 × CH), 126.0 (6 × CH), 128.2 (6 × CH), 157.6 (3 × C). The product was put under an argon atmosphere and transported into the glove box immediately.

### 3.9. Reactions of Potassium Triphenylmethanolate ***30*** at 40 °C

#### 3.9.1. Table 2, Entry 1

Potassium triphenylmethanolate **30** (597 mg, 2 mmol, 4.0 eq.), CBr_4_ (166 mg, 0.5 mmol, 1.0 eq.), adamantane **18** (68 mg, 0.5 mmol) and dichloromethane (3.13 mL) were added to an oven-dried pressure tube and the reaction mixture was stirred at 40 °C for 90 h in the dark. The reaction mixture was cooled to RT and quenched with aqueous hydrochloric acid (1 M, 5 mL) and extracted with diethyl ether (4 × 10 mL). The organic phases were combined, dried over Na_2_SO_4_, filtered and concentrated in vacuo. The yield of adamantane **18** (49%), 1-bromoadamantane **31** (7%), triphenylmethanol **32** (88%), benzophenone **33** (5%) and 4-benzhydrylphenol **34** (12%) were determined by adding 1,3,5-trimethoxybenzene to the crude mixture as an internal standard for ^1^H-NMR. The products were identified by the following characteristic signals; ^1^H-NMR (400 MHz, CDCl_3_) δ 1.75–1.78 (12 H, m), 1.88 (4 H, br s) for adamantane **18**; δ 1.74 (6 H, m), 2.12 (3 H, br s), 2.38 (6 H, m) for 1-bromoadamantane **31** [47]; δ 7.27–7.34 (15 H, m) for triphenylmethanol **32**; δ 7.49 (4 H, d, *J* = 8.0 Hz), 7.60 (2 H, d, *J* = 8.0 Hz), 7.82 (4 H, d, *J* = 8.0 Hz) for benzophenone **33** [51]; δ 5.49 (1 H, s), 6.73–6.77 (2 H, m), 6.97–7.00 (2 H, m) for 4-benzhydrylphenol **34** [52]; ^13^C-NMR (100 MHz, CDCl_3_) δ 28.3, 37.7 for adamantane **18**; δ 49.3, 35.5, 32.6 for 1-bromoadamantane **31**; δ 82.2, 127.4, 128.0, 147.0 for triphenylmethanol **32**; δ 56.1, 115.3, 144.3 for 4-benzhydroxylphenol **34**. These signals are consistent with the literature values and reference samples. This crude material was purified by column chromatography (0%–10% ethyl acetate in hexane) to give both benzophen-one **33** [51] (7 mg, 4%) as a yellow oil (Found: (GCMS-EI) C_13_H_10_O (M)^•+^ 182.0); *ν*_max_ (film)/cm^−1^ 3057, 1655, 1597, 1445, 1275, 1175, 939, 918, 808, 762; ^1^H-NMR (400 MHz, CDCl_3_) δ 7.49 (4 H, t, *J* = 8.0 Hz, Ar*H*), 7.60 (2 H, t, *J* = 8.0 Hz, Ar*H*), 7.82 (4 H, d, *J* = 8.0 Hz, Ar*H*); ^13^C-NMR (100 MHz, CDCl_3_) δ 128.2 (4 × CH), 130.2 (4 × CH), 132.6 (2 × CH), 137.5 (2 × C), 196.7 (C) and 4-benzhydrylphenol **34** [52] (18.9 mg, 7%) as a yellow oil (Found: (GCMS-EI) C_19_H_16_O (M)^•+^ 260.1); *ν*_max_(film)/cm^−1^ 3366, 2361, 2336, 1595, 1508, 1491, 1449, 1238, 1173, 1103, 1030, 816, 800, 750, 735; ^1^H-NMR (400 MHz, CDCl_3_) δ 4.82 (1 H, br s, O*H*), 5.49 (1 H, s, C*H*), 6.73–6.77 (2 H, m, Ar*H*), 6.97–7.00 (2 H, m, Ar*H*), 7.11–7.13 (4 H, m, Ar*H*), 7.19–7.32 (6 H, m, Ar*H*); ^13^C-NMR (100 MHz, CDCl_3_) δ 56.1 (CH), 115.3 (2 × CH), 126.4 (2 × CH), 128.4 (4 × CH), 129.5 (4 × CH), 130.7 (2 × CH), 136.4 (C), 144.3 (2 × C), 154.1 (C).

Triphenylmethanol **32** ^1^H-NMR (400 MHz, CDCl_3_) δ 2.79 (1 H, s, O*H*), 7.26–7.34 (15 H, m, Ar*H*), 7.57 (2 H, d, *J* = 8.4 Hz, Ar*H*); ^13^C-NMR (100 MHz, CDCl_3_) δ 82.2 (C), 127.4 (CH), 128.1 (6 × CH), 147.0 (9 × C). These signals are consistent with a commercial sample used as a reference.

#### 3.9.2. Table 2, Entry 2

Potassium triphenylmethanolate **30** (597 mg, 2 mmol, 4.0 eq.), adamantane **18** (68 mg, 0.5 mmol) and dichloromethane (3.13 mL) were added to an oven-dried pressure tube and the reaction mixture was stirred at 40 °C for 90 h in the dark. The reaction mixture was cooled to RT and quenched with aqueous hydrochloric acid (1 M, 5 mL) and extracted with diethyl ether (4 × 10 mL). The organic phases were combined, dried over Na_2_SO_4_, filtered and concentrated in vacuo. The yield of adamantane **18** (87%), triphenylmethanol **32** (81%) and benzophenone **33** (1%) were determined by adding 1,3,5-trimethoxybenzene to the crude mixture as an internal standard for ^1^H-NMR. The products were identified by the following characteristic signals; ^1^H-NMR (400 MHz, CDCl_3_) δ 1.75–1.78 (12 H, m), 1.88 (4 H, br s) for adamantane **18**; δ 7.27–7.34 (15 H, m) for triphenylmethanol **32**, δ 7.49 (4 H, d, *J* = 8.0 Hz, Ar*H*), 7.60 (2 H, d, *J* = 8.0 Hz, Ar*H*), 7.82 (4 H, d, *J* = 8.0 Hz, Ar*H*) for benzophenone **33**; ^13^C-NMR (100 MHz, CDCl_3_) δ 28.3, 37.7 for adamantane **18**; δ 82.2, 127.4, 128.0, 147.0 triphenylmethanol **32** [49]. These signals are consistent with the literature values and reference samples. 

## 4. Conclusions

**Scheme 7:**
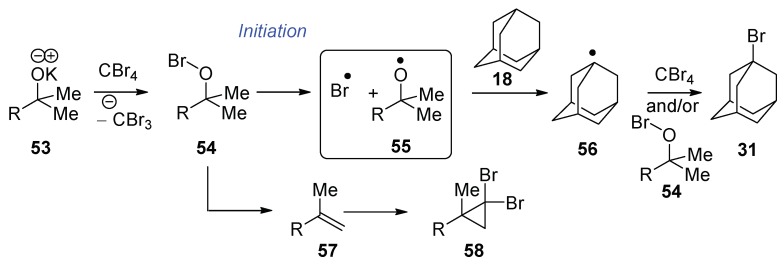
The modified mechanism for halogenation of adamantane.

This study has led to a revision of earlier thoughts on the mechanism for the halogenation of adamantane **18** by using a combination of KO*^t^*Bu and CBr_4_. It is proposed that the mechanism does not occur through SET, as was previously believed, but proceeds through hypobromite intermediates (Scheme 7). The alkoxide in the reaction mixture, **53**, forms a hypobromite in the presence of CBr_4_. The hypobromite **54** can undergo reaction along two pathways. The first option is an elimination reaction to form the alkene **57**, which reacts with carbenes formed in the reaction to afford final product **58**. The second pathway is the O–Br bond homolysis of the hypobromite **54**. This forms the alkoxyl radical **55** and a bromine radical. These radical intermediates perform a hydrogen atom abstraction from adamantane **18** to form adamantyl radical **56** (alternatively, the hydrogen atom abstraction may occur at the C-2 position to ultimately give the 2-Br isomer). The radical **56** will abstract a bromine from a hypobromite molecule **54**, or from CBr_4_, to form **31** and an alkoxyl radical **55**, or CBr_3_ radical. The radical formed will propagate the chain pathway by hydrogen atom abstraction from adamantane **18**, thus creating a radical chain mechanism. Thus, it appears that the search for reactions where ground-state KO*^t^*Bu behaves as an electron donor must continue. The outcomes of this project have led us to reexamine the remaining claims [3,4] of this phenomenon.

## Figures and Tables

**Table 1:**
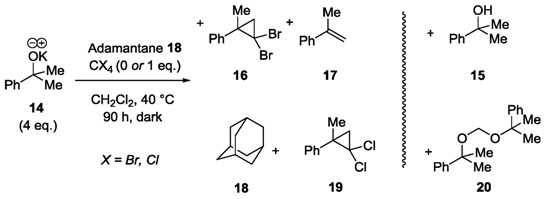
The reaction of the potassium 2-phenylpropan-2-olate 14 in dichloromethane at 40 °C ^a^.

Entry	Substrate 18 (mmol)/Additive (mmol)	16 (%)	17 (%)	18 (%)	19 (%)	15 (%)	20 (%)
		Yields Based on 0.5 mmol				Yields Based on [14] 2 mmol	
1	**18** (0.5)/CBr_4_ (0.5)	31	10	82	14	82	-
2	**18** (0.5)/CCl_4_ (0.5)	-	3	76	50	67	3
3	**18** (0.5)/-	-	2	90	-	55	24

^a^ All yields are calculated using 1,3,5-trimethoxybenzene as the internal standard (10 mol %) in ^1^H-NMR unless stated in the experimental information. As a precaution, reactions were conducted in foil-covered flasks. The yields of **16**–**19** were calculated using adamantane **18** as the limiting reagent (0.5 mmol). However, the yields of **15** and **20** were calculated based on the alkoxide **14** used (2 mmol).

**Table 2:**
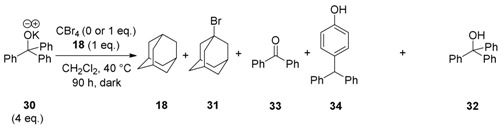
The reaction of potassium triphenylmethanolate **30** in dichloromethane at 40 °C ^a^.

Entry	Additive (mmol)	18 (%)	31 (%)	33 (%)	34 (%)	32 (%)
1	**18** (0.5) CBr_4_ (0.5)	49	7	5	12	88
2	**18** (0.5)	87	0	1	0	81

^a^ All yields were calculated using 1,3,5-trimethoxybenzene as the internal standard (10 mol %) in the ^1^H-NMR unless stated in the experimental information. The yields of **18**, **31**, **33**, **34** were calculated using adamantane **18** as the limiting reagent. However, the yield of **32** was calculated based on the alkoxide **30**.

## Supplementary Materials

The following are available online, (i)Additional computational analysis and xyz coordinates relating to all computed structures (ii) Table S1 “The reaction of *tert*-butyl hypochlorite 46 in dichloromethane or carbon tetrachloride as solvent” (iii) Table S2. *tert*-Butyl hypobromite 50 in bromination of adamantane 18; (iv) Experimental procesdures and spectroscopid data in support of the experiments discussed in those Tables (v) NMR spectra of key products.
