# Supplementary material for: KOtBu as a Single Electron Donor? Revisiting the Halogenation of Alkanes with CBr4 and CCl4
Source: Molecules. 2018 May 1;23(5):1055. doi: 10.3390/molecules23051055 (PMC6102552; doi:10.3390/molecules23051055)
Supplement: Supplementary file 1 [file molecules-23-01055-s001.pdf]

KO*t*Bu as a single electron donor? Revisiting the halogenation of alkanes with CBr_4 and CCl_4

Katie J. Emery, Allan Young, J. Norman Arokianathar, Tell Tuttle, and John A. Murphy

SUPPORTING INFORMATION

Computational analysis of the reactivity of methylstyrene 17 in dichloromethane.

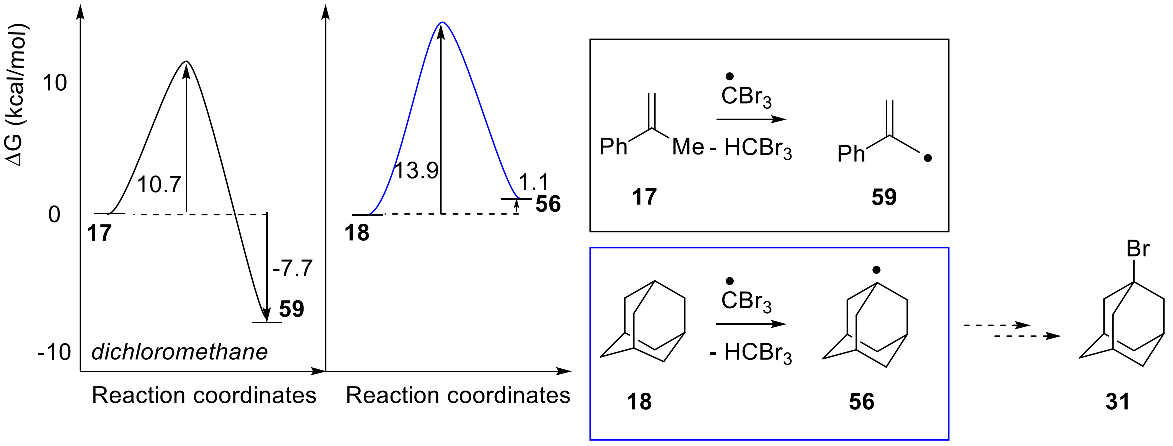

Computational modelling of single electron transfer from alkoxides, 14 and 29, to CBr_4 in dichloromethane or CCl_4 in tetrachloromethane.

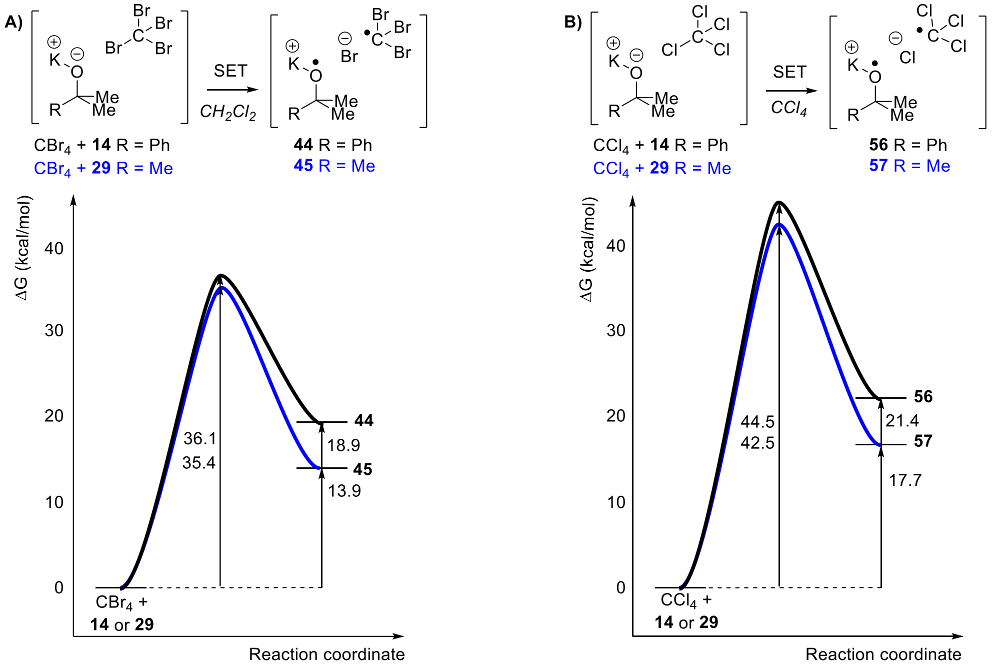

Figure S1. Energy profile for SET from potassium 2-phenylpropan-2-olate **14** (black line) or KO^{t}Bu **29** (blue line) to **A)** CBr_4 in dichloromethane (left) and **B)** CCl_4 in CCl_4 (right).

The energy barriers for SET from either KO^{t}Bu, **29**, or potassium 2-phenylpropan-2-olate **14** to a molecule of CBr_4 were calculated to be $\Delta G^‡$ = 35.4 kcal/mol and 36.1 kcal/mol respectively (SET from KO^{t}Bu to CCl_4 in dichloromethane: $\Delta G^‡$ = 38.8 kcal/mol and ΔG_{rxn} = 16.0 kcal/mol), and these barriers are not accessible for a reaction performed at 40 °C. When the reaction was performed in CCl_4 a similar energy profile was obtained for the SET from either of the two alkoxides to a molecule of CCl_4. The energy barriers calculated were $\Delta G^‡$ = 42.5 kcal/mol and 44.5 kcal/mol for SET to CCl_4 from KO^{t}Bu **29** and potassium 2-phenylpropan-2-olate **14** respectively. From these computationally derived energy profiles for the SET step, it suggests that the initiation for this halogenation of adamantane **18** is not *via* SET from the alkoxide.

To reassure ourselves that our proposed pathway (Scheme 7) can occur experimentally under our conditions, *tert*-butyl hypochlorite **46** was prepared and subjected to our reaction conditions (Table S1).The *tert*-butyl hypochlorite **46** chlorinated adamantane **18**, in both dichloromethane and CCl_4 as the solvents, to give products: 1-chloroadamantane **47** (25% and 11% respectively), and 1,3-dichloroadamantane **48** (16% and 26% respectively) (Table S1, entry 1 and 2). 2-Chloroadamantane **49** (13%) was formed in dichloromethane, but not in CCl_4. We also explored whether an efficient chain reaction could be initiated by lower amounts of *tert*-butyl hypochlorite **46** when CCl_4 was added in stoichiometric quantities (Table S1, entries 3-5).

Table S1. The reaction of *tert*-butyl hypochlorite **46** in dichloromethane or carbon tetrachloride as solvent.

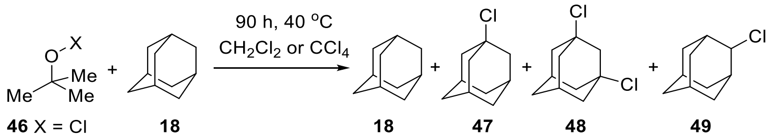

Entry[a]	Substrate (mmol)	Additive (mmol)	Solvent (mL)	18 (%)	47 (%)	48 (%)	49 (%)
1	**46** (2)	**18** (0.5)	CH_2Cl_2 (3.13)	10	25	16	13
2	**46** (2)	**18** (0.5)	CCl_4 (3.13)	2	11	26	0
3	**46** (0.4)	**18** (0.5) CCl_4 (0.5)	CH_2Cl_2 (3.13)	58	12	1	6
4	**46** (0.1)	**18** (0.5) CCl_4 (0.5)	CH_2Cl_2 (3.13)	66	<1		
5		**18** (0.5) CCl_4 (0.5)	CH_2Cl_2 (3.13)	71			

[a] yields were determined by spiking with 1,3,5-trimethoxybenzene (internal standard, 8.4mg, 10mol%)

Table S2. *tert*-Butyl hypobromite **50 in** bromination of adamantane **18**.

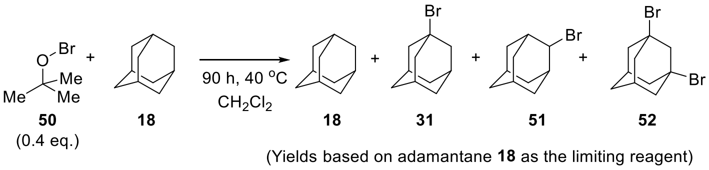

Entry[a]	Substrate (mmol)	Additive (mmol)	Solvent (mL)	18 (%)	31 (%)	51 (%)	52 (%)
1	**50** (0.4)	**18** (0.5)	CH_2Cl_2 (3.13)	70	16	4	<1
2	**50** (0.4)	**18** (0.5) CBr_4 (0.5)	CH_2Cl_2 (3.13)	2	28	5	18
3	**50** (0.1)	**18** (0.5) CBr_4 (0.5)	CH_2Cl_2 (3.13)	29	35	14	11
4		**18** (0.5) CBr_4 (0.5)	CH_2Cl_2 (3.13)	75			

[a] yields were determined by spiking with 1,3,5-trimethoxybenzene (internal standard, 8.4mg, 10mol%)

In addition, *tert*-butyl hypobromite **50** was synthesised and subjected to similar reaction conditions (Scheme 7), affording analogous bromoadamantanes - (adamantane **18** (70%), 1-bromooadamantane **31** (16%), 2-bromooadamantane **51** (4%), and 1,3-dibromooadamantane **48** (<1%). We also explored whether an efficient chain reaction could be initiated by lower amounts of *tert*-butyl hypbromite **46** when CBr_4 was added (Table S2, entries 2-4). In general, these conditions were not conducive to efficient halogenations.[1]

Preparation of tert-butyl hypochlorite ***46***

Throughout the experiment, all the equipment was covered in aluminium foil and the reaction mixture was always kept in the dark. NaOCl (0.6 M, 200 mL, 1.4 eq.) was added to a round-bottomed flask and the reaction mixture was stirred at 0 °C for 10 min. A mixture of *tert*-butanol (8 mL, 84 mmol) and acetic acid (5.3 mL, 92 mmol, 1.11 eq.) were added in one batch under vigorous stirring and the reaction mixture was stirred at 0 °C for 10 min. The reaction mixture phase-separated. The top yellow phase was separated from the reaction mixture, washed with saturated aqueous $NaHCO_3$ solution (50 mL) and water (50 mL), dried over Na_2SO_4 to give *tert*-butyl hypochlorite **46** [2,3] (0.82 mL, d = 1.128 g mL^{-1}, 34%) as a yellow oil ^{1}H-NMR (400 MHz, $CDCl_3$) δ 1.32 (9 H, s, 3 x C*H*$_3$); ^{13}C-NMR (100 MHz, $CDCl_3$) δ 26.9 (3 x CH_3), 84.0 (C). These signals are consistent with the literature values. The *tert*-butyl hypochlorite **46** was used immediately.

Reactions of tert-butyl hypochlorite 46 with adamantane ***18:***

Table S1, entry 1

tert-Butyl hypochlorite **46** (0.19 mL, freshly prepared, 2 mmol, 4.0 eq.), adamantane **18** (68 mg, 0.5 mmol) and dichloromethane (3.13 mL) were added to an oven-dried pressure tube and the reaction mixture was stirred at 40 °C for 90 h in the dark. The reaction mixture was cooled to RT and quenched with aqueous hydrochloric acid (1 M, 5 mL) and extracted with dichloromethane (4 x 10 mL). The organic phases were combined, dried over Na_2SO_4, filtered and concentrated *in vacuo*. The yield of adamantane **18** (10%), 1-chloroadamantane **47** (25%), 1,3-dichloroadamantane **48** (16%) and 2-chloroadamantane **49** (13%) were determined by adding 1,3,5-trimethoxybenzene to the crude mixture as an internal standard for ^{1}H-NMR. The products were identified by the following characteristic signals; ^{1}H-NMR (400 MHz, $CDCl_3$) δ 1.75 – 1.78 (12 H, m), 1.88 (4 H, br s) for adamantane **18**; δ 1.68 (6 H, s), 2.14 (9 H, s) for 1-chloroadamantane **47**;[4] δ 2.06 (8 H, d, *J* = 4 Hz), 2.47 (2 H, s) for 1,3-chloroadamantane **48**;[5] δ 4.40 (1 H, s) 2-chloroadamantane **49**;[6] ^{13}C-NMR (100 MHz, $CDCl_3$) δ 28.5, 37.9 for adamantane **18**; δ 31.9, 35.7, 47.9 for 1-chloroadamantane **47**;[4] δ 33.5, 33.8, 45.9, 56.6, 66.9 for 1,3-dichloroadamantane **48**;[5,6] δ 26.9, 27.5, 31.1, 35.9, 37.8, 38.3, 68.4 2-chloroadamantane **49**. Analysis of the crude material showed that the products were inseparable, hence the analysis of the product mixture was performed using experimental ^{1}H-NMR and ^{13}C-NMR values reported within the literature as a reference.

1-Chloroadamantane **47**[55] ^{1}H-NMR (400 MHz, $CDCl_3$) δ 1.68–1.69 (6 H, m, 6 x C*H*$_2$), 2.14 (9 H, s, 3 x C*H* and 3 x C*H*$_2$); ^{13}C-NMR (100 MHz, $CDCl_3$) δ 31.9, 35.7, 47.9, 69.1.

1,3-Dichloroadamantane **48** [5,6] ^{1}H-NMR (400 MHz, $CDCl_3$) δ 1.63 (2 H, br s, C*H*$_2$), 2.06 (8 H, d, 4 x C*H*$_2$), 2.30 (2 H, br s, 2 x C*H*), 2.46 (2 H, s, C*H*$_2$); ^{13}C-NMR (100 MHz, $CDCl_3$) δ 33.4 (CH_2), 33.7, (2 x CH), 45.7 (4 x CH_2), 56.5 (CH_2), 66.7 (2 x C).

2-Chloroadamantane **49**[6] ^{1}H-NMR (500 MHz, $CDCl_3$) δ 1.56–1.59 (2 H, m, C*H*$_2$), 1.76–1.87 (6 H, m, 2 x C*H* and 2 x C*H*$_2$), 1.93–1.96 (2 H, m, C*H*$_2$), 2.08 (2 H, br s, 2 x C*H*), 2.26–2.29 (2 H, m, C*H*$_2$), 4.40 (1 H, s, C*H*); ^{13}C-NMR (125 MHz, $CDCl_3$) δ 27.0, 27.6, 31.2, 36.0, 37.9, 38.3, 68.5.

Table S1, entry 2

tert-Butyl hypochlorite **46** (0.19 mL, freshly prepared, 2 mmol, 4.0 eq.), adamantane **18** (68 mg, 0.5 mmol) and CCl_4 (3.13 mL) were added to an oven-dried pressure tube and the reaction mixture was stirred at 40 °C for 90 h in the dark. The reaction mixture was cooled to RT and quenched with aqueous hydrochloric acid (1 M, 5 mL) and extracted with dichloromethane (4 x 10 mL). The organic phases were combined, dried over Na_2SO_4, filtered and concentrated *in vacuo*. The yield of adamantane **18** (2%), 1-chloroadamantane **47** (11%) and 1,3-

dichloroadamantane **48** (26%) were determined by adding 1,3,5-trimethoxybenzene to the crude mixture as an internal standard for ^{1}H-NMR. The products were identified by the following characteristic signals; ^{1}H-NMR (400 MHz, $CDCl_3$) δ 1.75–1.78 (12 H, m), 1.88 (4 H, br s) for adamantane **18**; δ 1.68 (6 H, s), 2.14 (9 H, s) for 1-chloroadamantane **47**;[4] δ 2.06 (8 H, d, *J* = 4.0 Hz), 2.47 (2 H, s) for 1,3-chloroadamantane **48**;[5] ^{13}C-NMR (100 MHz, $CDCl_3$) δ 28.5, 37.9 for adamantane **18**; δ 31.9, 35.7, 47.9 for 1-chloroadamantane **47**;[7] δ 33.5, 33.8, 45.9, 56.6, 66.9 for 1,3-chloroadamantane **48**.[5] These signals are consistent with the literature values and reference samples.

Table S1, entry 3

tert-Butyl hypochlorite **46** (24 mg, freshly prepared, 0.22 mmol, 0.4 eq.), adamantane **18** (68 mg, 0.5 mmol), carbon tetrachloride (77 mg, 0.5 mmol, 1 eq.) and dichloromethane (3.13 mL) were added to an oven-dried pressure tube and the reaction mixture was stirred at 40 °C for 90 h in the dark. The reaction mixture was cooled to RT and quenched with aqueous hydrochloric acid (1 M, 5 mL) and extracted with dichloromethane (4 x 10 mL). The organic phases were combined, dried over Na_2SO_4, filtered and concentrated *in vacuo*. The yield of adamantane **18** (58%), 1-chloroadamantane **47** (12%), 1,3-dichloroadamantane **48** (1%) and 2-chloroadamantane **49** (6%) were determined by adding 1,3,5-trimethoxybenzene to the crude mixture as an internal standard for ^{1}H-NMR. The products were identified by the following characteristic signals; ^{1}H-NMR (400 MHz, $CDCl_3$) δ 1.75 – 1.78 (12 H, m), 1.88 (4 H, br s) for adamantane **18**; δ 1.68 (6 H, s), 2.14 (9 H, s) for 1-chloroadamantane **47**;[7] δ 2.06 (8 H, d, *J* = 4 Hz), 2.47 (2 H, s) for 1,3-chloroadamantane **48**;[2] δ 4.40 (1 H, s) 2-chloroadamantane **49**.[9]

Analysis of the crude material showed that the products were inseparable, hence the analysis of the product mixture was performed using experimental ^{1}H-NMR and ^{13}C-NMR values reported within the literature as a reference.

1-Chloroadamantane **47**[4] ^{1}H-NMR (400 MHz, $CDCl_3$) δ 1.68 – 1.69 (6 H, m, 6 x C*H*$_2$), 2.14 (9 H, s, 3 x C*H* and 3 x C*H*$_2$); ^{13}C-NMR (100 MHz, $CDCl_3$) δ 31.9, 35.7, 47.9, 69.1.

1,3-Dichloroadamantane **48**[5,8] ^{1}H-NMR (400 MHz, $CDCl_3$) δ 1.63 (2 H, br s, C*H*$_2$), 2.06 (8 H, d, 4 x C*H*$_2$), 2.30 (2 H, br s, 2 x C*H*), 2.46 (2 H, s, C*H*$_2$); ^{13}C-NMR (100 MHz, $CDCl_3$) δ 33.4 (CH_2), 33.7, (2 x CH), 45.7 (4 x CH_2), 56.5 (CH_2), 66.7 (2 x C).

2-Chloroadamantane **49**[6] ^{1}H-NMR (500 MHz, $CDCl_3$) δ 1.56 – 1.59 (2 H, m, C*H*$_2$), 1.76 – 1.87 (6 H, m, 2 x C*H* and 2 x C*H*$_2$), 1.93 – 1.96 (2 H, m, C*H*$_2$), 2.08 (2 H, br s, 2 x C*H*), 2.26 – 2.29 (2 H, m, C*H*$_2$), 4.40 (1 H, s, C*H*); ^{13}C-NMR (125 MHz, $CDCl_3$) δ 27.0, 27.6, 31.2, 36.0, 37.9, 38.3, 68.5.

Table S1, entry 4

tert-Butyl hypochlorite **46** (5.4 mg, freshly prepared, 0.05 mmol, 0.1 eq.), adamantane **18** (68 mg, 0.5 mmol), carbon tetrachloride (77 mg, 0.5 mmol, 1 eq.) and dichloromethane (3.13 mL) were added to an oven-dried pressure tube and the reaction mixture was stirred at 40 °C for 90 h in the dark. The reaction mixture was cooled to RT and quenched with aqueous hydrochloric acid (1 M, 5 mL) and extracted with dichloromethane (4 x 10 mL). The organic phases were combined, dried over Na_2SO_4, filtered and concentrated *in vacuo*. The yield of adamantane **18** (66%) and 1-chloroadamantane **47** (<1%) were determined by adding 1,3,5-trimethoxybenzene to the crude mixture as an internal standard for ^{1}H-NMR. The products were identified by the following characteristic signals; ^{1}H-NMR (400 MHz, $CDCl_3$) δ 1.75 – 1.78 (12 H, m), 1.88 (4 H, br s) for adamantane **18**; δ 1.68 (6 H, s), 2.14 (9 H, s) for 1-chloroadamantane **47**;[4] ^{13}C NMR (100MHz, $CDCl_3$) δ 28.5, 37.9 for adamantane **18**; δ 31.9, 35.7, 47.9 for 1-chloroadamantane **47**.[4]

Analysis of the crude material showed that the products were inseparable, hence the analysis of the product mixture was performed using experimental ^{1}H-NMR and ^{13}C-NMR values reported within the literature as a reference.

1-Chloroadamantane **47**[7] ^{1}H-NMR (400 MHz, $CDCl_3$) δ 1.68 – 1.69 (6 H, m, 6 x C*H*$_2$), 2.14 (9 H, s, 3 x C*H* and 3 x C*H*$_2$); ^{13}C-NMR (100 MHz, $CDCl_3$) δ 31.9, 35.7, 47.9, 69.1.

Table S1, entry 5, blank reaction

Adamantane **18** (68 mg, 0.5 mmol), carbon tetrachloride (77 mg, 0.5 mmol, 1 eq.) and dichloromethane (3.13 mL) were added to an oven-dried pressure tube and the reaction mixture was stirred at 40 °C for 90 h in the dark. The reaction mixture was cooled to RT and quenched with aqueous hydrochloric acid (1 M, 5 mL) and extracted with dichloromethane (4 x 10 mL). The organic phases were combined, dried over Na_2SO_4, filtered and concentrated *in vacuo*. The yield of adamantane **18** (71%) was determined by adding 1,3,5-trimethoxybenzene to the crude mixture as an internal standard for ^{1}H-NMR. The product was identified by the following characteristic signals; ^{1}H-NMR (400 MHz, $CDCl_3$) δ 1.75 – 1.78 (12 H, m), 1.88 (4 H, br s) for adamantane **18**. ^{13}C NMR (100MHz, $CDCl_3$) δ 28.5, 37.9. These signals are consistent with the literature values and reference samples.

*Reaction of KO^{t}Bu with adamantane **18** in CCl_4*

KO^{t}Bu **29** (224 mg, 2 mmol, 4.0 eq.), adamantane **18** (68 mg, 0.5 mmol) and CCl_4 (3.13 mL) were added to an oven-dried pressure tube and the reaction mixture was stirred at 40 °C for 90 h in the dark. The reaction mixture was cooled to RT and quenched with aqueous hydrochloric acid (1 M, 5 mL) and extracted with dichloromethane (4 x 10 mL). The organic phases were combined, dried over Na_2SO_4, filtered and concentrated *in vacuo*. The yield of adamantane **18** (64%) and 1-chloroadamantane **47**[4] (5%) were determined by adding 1,3,5-trimethoxybenzene to the crude mixture as an internal standard for ^{1}H-NMR. The products were identified by the following characteristic signals; ^{1}H-NMR (400 MHz, $CDCl_3$) δ 1.75–1.78 (12 H, m), 1.88 (4 H, br s) for adamantane **18**; δ 1.68 (6 H, s), 2.14 (9 H, s) for 1-chloroadamantane **47**;[4] ^{13}C-NMR (100 MHz, $CDCl_3$) δ 28.5, 37.9 for adamantane **18**; δ 31.7, 35.6, 47.8 for 1-chloroadamantane **47**.[4] These signals are consistent with the literature values and reference samples.

*Synthesis of tert-butyl hypobromite **50***

Throughout the experiment all the equipment was covered in aluminium foil and the reaction mixture was always kept in the dark. To a round-bottomed flask was added NaOCl (0.6 M, 200 mL, 1.4 eq.) and the reaction mixture was stirred at 0 °C for 10 min. NaBr (9.26 g, 90 mmol, 1.1 eq.) was added and the reaction mixture was stirred at 0 °C for 2 min. A mixture of *tert*-butanol (8 mL, 84 mmol) and acetic acid (5.3 mL, 92 mmol, 1.11 eq.) was added in one batch under vigorous stirring and the reaction mixture was stirred at 0 °C for 1 h. The reaction mixture was extracted with dichloromethane (10 mL), washed with water (100 mL), dried over Na_2SO_4 to give *tert*-butyl hypobromite **50**[57] as a dark red solution in dichloromethane ^{1}H-NMR (400 MHz, $CDCl_3$) δ 1.27 (9 H, s, 3 x C*H*$_3$); ^{13}C-NMR (100 MHz, $CDCl_3$) δ 27.5 (3 x CH_3), 82.9 (C). The *tert*-butyl hypobromite **50** was used immediately.

To quantify the amount of *tert*-butyl hypobromite **50** formed, 0.2 mL of the *tert*-butyl hypobromite/dichloromethane solution was transferred to a small vial and a solution of 1,2-dibromoethane (internal standard) in dichloromethane (1 mL, 0.5 M, 0.5 mmol) was added. The concentration was determined from ^{1}H-NMR: In 0.2 mL of the *tert*-butyl hypobromite / dichloromethane solution, there is 0.052 mmol of *tert*-butyl hypobromite **50**. The compound was used immediately in reactions. To ensure the *tert*-butyl hypobromite **50** had not decomposed prior to being used in the reactions, the *tert*-butyl hypobromite / dichloromethane solution was analysed in the same way immediately after being used, to determine the amount of *tert*-butyl hypobromite **50** present in the *tert*-butyl hypobromite/dichloromethane solution: the amount was determined from ^{1}H-NMR: In 0.2 mL of the *tert*-butyl hypobromite/dichloromethane solution, there was 0.052 mmol of *tert*-butyl hypobromite **50**.

*Reaction of tert-butyl hypobromite **50** with adamantane **18**.*

tert-Butyl hypobromite **50** (0.87 mL, freshly prepared and used immediately, 0.22 mmol, 0.4 eq.), adamantane **18** (68 mg, 0.5 mmol) and dichloromethane (3.13 mL) were added to an oven-dried pressure tube and the reaction mixture was stirred at 40 °C for 90 h in the dark. The reaction mixture was cooled to RT and quenched with aqueous hydrochloric acid (1 M, 5 mL) and extracted with dichloromethane (4 x 10 mL). The organic phases were combined, dried over Na_2SO_4, filtered and concentrated *in vacuo*. The yield of adamantane **18** (70%), 1-bromoadamantane **31** (16%), 2-bromoadamantane **51** (4%) and 1,3-

bromoadamantane **52** (< 1%) were determined by adding 1,3,5-trimethoxybenzene to the crude mixture as an internal standard for ^{1}H-NMR. The products were identified by the following characteristic signals; ^{1}H-NMR (400 MHz, $CDCl_3$) δ 1.74–1.76 (12 H, m), 1.88 (4 H, br s) for adamantane **18**; δ 1.72 (6 H, m), 2.10 (3 H, br s), 2.36 (6 H, m) for 1-bromoadamantane **31**;[19] δ 1.97–2.00 (2 H, m), 2.15 (2 H, br s), 2.33 (2 H, br s), 4.68 (1 H, br s) for 2-bromoadamantane **51**;[48] δ 1.70 (2 H, m), 2.25–2.30 (10 H, m), 2.87 (2 H, br s) for 1,3-dibromoadamantane **52**.[47] ^{13}C-NMR (100 MHz, $CDCl_3$) δ 28.5, 37.9 adamantane **18**; δ 32.8, 35.7, 49.5 for 1-bromoadamantane **31**. These signals are consistent with the literature values and reference samples.

*Reactions of KO^{t}Bu (**29**) with adamantane (**18**) and CBr_4*

Table S2, entry 1

KO^{t}Bu **29** (224 mg, 2 mmol, 4.0 eq.), adamantane **18** (68 mg, 0.5 mmol), CBr_4 (166 mg, 0.5 mmol, 1.0 eq.) and dichloromethane (3.13 mL) were added to an oven-dried pressure tube and the reaction mixture was stirred at 40 °C for 90 h in the dark. The reaction mixture was cooled to RT and quenched with aqueous hydrochloric acid (1 M, 5 mL) and extracted with dichloromethane (4 x 10 mL). The organic phases were combined and dried over Na_2SO_4, filtered and concentrated *in vacuo*. The yield of adamantane **18** (84%) and 1-bromoadamantane **31** (4%) were determined by adding 1,3,5-trimethoxybenzene to the crude mixture as an internal standard for ^{1}H-NMR. The products were identified by the following characteristic signals; ^{1}H-NMR (400 MHz, $CDCl_3$) δ 1.74–1.76 (12 H, m), 1.88 (4 H, br s) for adamantane **18**; δ 1.72 (6 H, m), 2.10 (3 H, br s), 2.36 (6 H, m) for 1-bromoadamantane **31**;[11] ^{13}C NMR (100 MHz, $CDCl_3$) δ 28.5, 37.9 adamantane **18**; δ 32.8, 35.7, 49.5 for 1-bromoadamantane **31**. These signals are consistent with the literature values and reference samples.[6, 11]

Table S2, entry 2

tert-Butyl hypobromite **50** (1.89 mL, freshly prepared and used immediately, 0.22 mmol, 0.4 eq.), adamantane **18** (68 mg, 0.5 mmol), carbon tetrabromide (166 mg, 0.5 mmol, 1 eq.) and dichloromethane (3.13 mL) were added to an oven-dried pressure tube and the reaction mixture was stirred at 40 °C for 90 h in the dark. The reaction mixture was cooled to RT and quenched with aqueous hydrochloric acid (1 M, 5 mL) and extracted with dichloromethane (4 x 10 mL). The organic phases were combined, dried over Na_2SO_4, filtered and concentrated *in vacuo*. The yield of adamantane **18** (9%), 1-bromoadamantane **31** (28%), 2-bromoadamantane **51** (5%) and 1,3-bromoadamantane **52** (18%) were determined by adding 1,3,5-trimethoxybenzene to the crude mixture as an internal standard for ^{1}H-NMR. The products were identified by the following characteristic signals; ^{1}H-NMR (400 MHz, $CDCl_3$) δ 1.74 – 1.76 (12 H, m), 1.88 (4 H, br s) for adamantane **18**; δ 1.72 (6 H, m), 2.10 (3 H, br s), 2.36 (6 H, m) for 1-bromoadamantane **31**;[1] δ 1.97 – 2.00 (2 H, m), 2.15 (2 H, br s), 2.33 (2 H, br s), 4.68 (1 H, br s) for 2-bromoadamantane **51**;[6] δ 1.70 (2 H, m), 2.25 – 2.30 (10 H, m), 2.87 (2 H, br s) for 1,3-dibromoadamantane **52**.[11] ^{13}C NMR (100MHz, $CDCl_3$) δ 28.5, 37.9 for adamantane **18**; δ 32.8, 35.7, 49.5 for 1-bromoadamantane **31**. These signals are consistent with the literature values and reference samples.

Table S2, entry 3

tert-Butyl hypobromite **50** (0.33 mL, freshly prepared and used immediately, 0.05 mmol, 0.1 eq.), adamantane **18** (68 mg, 0.5 mmol), carbon tetrabromide (166 mg, 0.5 mmol, 1 eq.) and dichloromethane (3.13 mL) were added to an oven-dried pressure tube and the reaction mixture was stirred at 40 °C for 90 h in the dark. The reaction mixture was cooled to RT and quenched with aqueous hydrochloric acid (1 M, 5 mL) and extracted with dichloromethane (4 x 10 mL). The organic phases were combined, dried over Na_2SO_4, filtered and concentrated *in vacuo*. The yield of adamantane **18** (29%), 1-bromoadamantane **31** (35%), 2-bromoadamantane **51** (14%) and 1,3-bromoadamantane **52** (11%) were determined by adding 1,3,5-trimethoxybenzene to the crude mixture as an internal standard for ^{1}H-NMR. The products were identified by the following characteristic signals; ^{1}H-NMR (400 MHz, $CDCl_3$) δ 1.74 – 1.76 (12 H, m), 1.88 (4 H, br s) for adamantane **18**; δ 1.72 (6 H, m), 2.10 (3 H, br s), 2.36 (6 H, m) for 1-bromoadamantane **31**;[12] δ 1.97 – 2.00 (2 H, m), 2.15 (2 H, br s), 2.33 (2 H, br s), 4.68 (1 H, br s) for 2-bromoadamantane **51**;[45] δ 1.70 (2 H, m), 2.25 – 2.30 (10 H, m), 2.87 (2 H, br s) for 1,3-dibromoadamantane **52**.[13] ^{13}C NMR (100MHz, $CDCl_3$) δ 28.5, 37.9 for adamantane **18**; δ 32.8, 35.7, 49.5 for 1-bromoadamantane **31**. These signals are consistent with the literature values and reference samples.

Table S2, entry 4, blank reaction

Adamantane **18** (68 mg, 0.5 mmol), carbon tetrabromide (166 mg, 0.5 mmol, 1 eq.) and dichloromethane (3.13 mL) were added to an oven-dried pressure tube and the reaction mixture was stirred at 40 °C for 90 h in the dark. The reaction mixture was cooled to RT and quenched with aqueous hydrochloric acid (1 M, 5 mL) and extracted with dichloromethane (4 x 10 mL). The organic phases were combined, dried over Na_2SO_4, filtered and concentrated *in vacuo*. The yield of adamantane **18** (75%) was determined by adding 1,3,5-trimethoxybenzene to the crude mixture as an internal standard for ^{1}H-NMR. The product was identified by the following characteristic signals; ^{1}H-NMR (400 MHz, $CDCl_3$) δ 1.74 – 1.76 (12 H, m), 1.88 (4 H, br s) for adamantane **18**. ^{13}C NMR (100MHz, $CDCl_3$) δ 28.5, 37.9. These signals are consistent with the literature values and reference samples.

References

1. For efficient halogenation of alkanes with hypohalites, seeR. Montoro and T. Wirth, *Synthesis*, 2005, 1473–1478.
2. G. Bentzinger, W. De Souza, C. Mullié, P. Agnamey, A. Dassonville-Klimpt, P. Sonnet, *Tetrahedron: Asymmetry*, 2016, **27**, 1–11.
3. F. Kleinbeck, G. J. Fettes, L. D. Fader, E. M. Carreira, *Chem. Eur. J.*, 2012, **18**, 3598–3610.
4. L. Candish, E. A. Standley, A. Gómez-Suárez, S. Mukherjee, F. Glorius, *Chem. Eur. J.*, 2016, **22**, 9971–9974.
5. J. Y. Becker, S. Yatziv, *J. Org. Chem.*, 1988, **53**, 1744–1748.
6. Y. Liu, Y. Xu, S. H. Jung, J. Chae, *Synlett*, 2012, **23**, 2692–2698.
7. Y. Sato, T. Aoyama, T. Takido, M. Kodomari, *Tetrahedron*, 2012, **68**, 7077–7081.
8. R. I. Khusnutdinov, N. A. Shchadneva, A. R. Bayguzina, T. Oshnyakova, Y. Y. Mayakova, U. M. Dzhemilev, *Russ. J. Org.Chem.*, 2013, **49**, 1557–1566.
9. M. J. Frisch, G. W. Trucks, H. B. Schlegel, G. E. Scuseria, M. A. Robb, J. R. Cheeseman, G. Scalmani, V. Barone, B. Mennucci, G. A. Petersson, H. Nakatsuji, M. Caricato, X. Li, H. P. Hratchian, A. F. Izmaylov, J. Bloino, G. Zheng, J. L. Sonnenberg, M. Hada, M. Ehara, K. Toyota, R. Fukuda, J. Hasegawa, M. Ishida, T. Nakajima, Y. Honda, O. Kitao, H. Nakai, T. Vreven, J. A. Montgomery Jr., J. E. Peralta, F. Ogliaro, M. J. Bearpark, J. Heyd, E. N. Brothers, K. N. Kudin, V. N. Staroverov, R. Kobayashi, J. Normand, K. Raghavachari, A. P. Rendell, J. C. Burant, S. S. Iyengar, J. Tomasi, M. Cossi, N. Rega, N. J. Millam, M. Klene, J. E. Knox, J. B. Cross, V. Bakken, C. Adamo, J. Jaramillo, R. Gomperts, R. E. Stratmann, O. Yazyev, A. J. Austin, R. Cammi, C. Pomelli, J. W. Ochterski, R. L. Martin, K. Morokuma, V. G. Zakrzewski, G. A. Voth, P. Salvador, J. J. Dannenberg, S. Dapprich, A. D. Daniels, Ö. Farkas, J. B. Foresman, J. V. Ortiz, J. Cioslowski, D. J. Fox *Gaussian 09*, Gaussian, Inc.: Wallingford, CT, USA, 2009
10. C. Walling, J. A. McGuinness, *J. Am. Chem. Soc.*, 1969, **91**, 2053–2058.
11. S. E. Denmark, B. R. Henke, *J. Am. Chem. Soc.*, 1991, **113**, 2177–2194
12. A. A. Fokin and P. R. Schreiner, *Adv. Synth. Catal.*, 2003, **345**, 1035–1052.
13. 44. M. Cossi, N. Rega, G. Scalmani, V. Barone, *J. Comput. Chem.*, 2003, **24**, 669–681

^{1}H and ^{13}C-NMR Spectra

^{1}H-NMR and ^{13}C-NMR 14

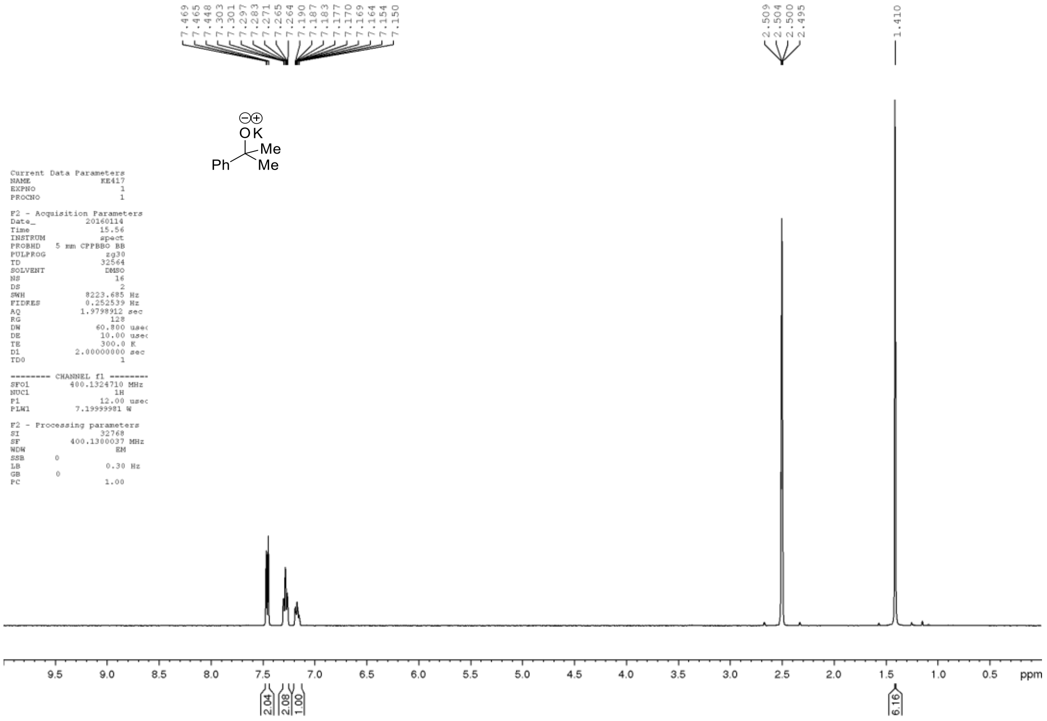

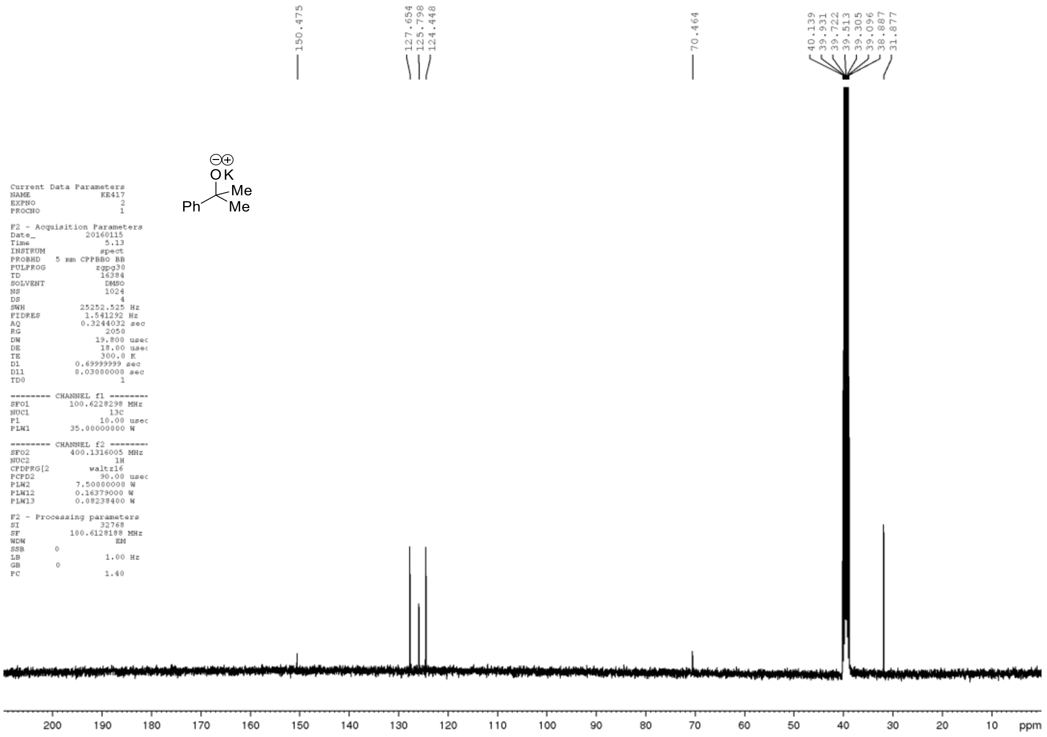

^{1}H-NMR and ^{13}C-NMR 17 (Commercial sample used as a reference)

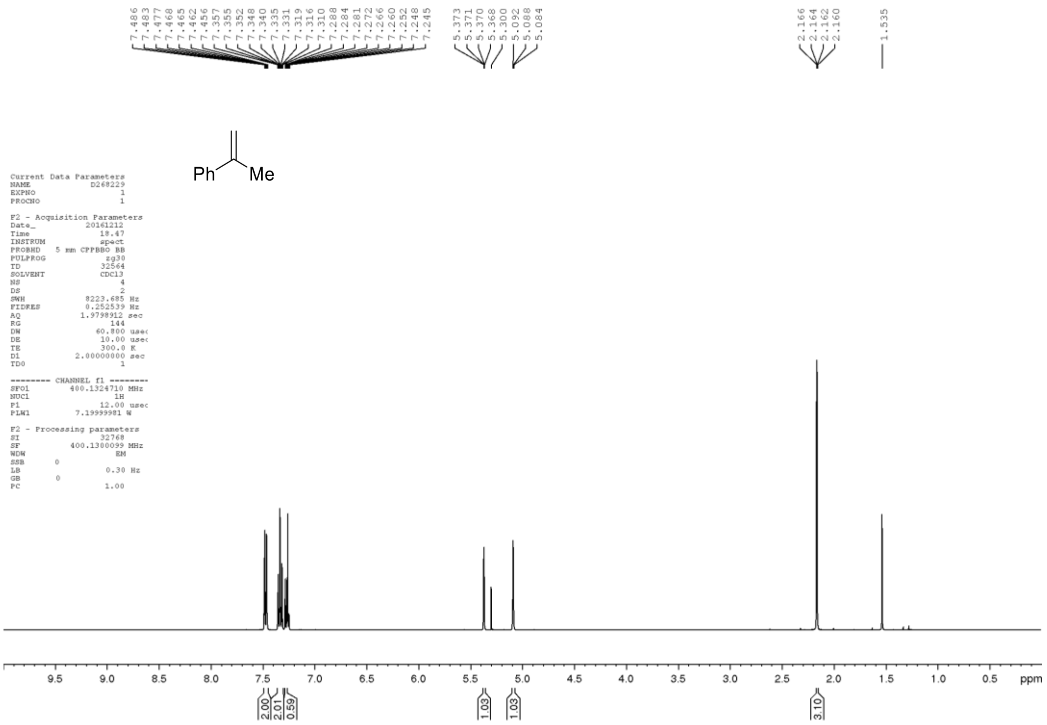

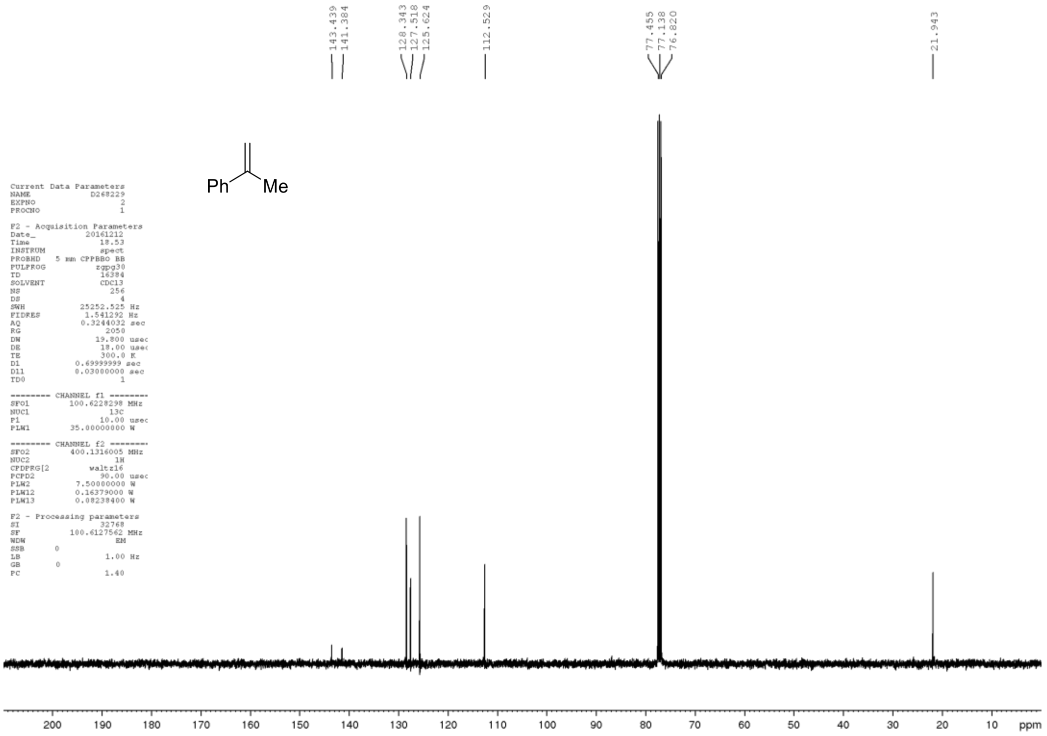

^{1}H-NMR and ^{13}C-NMR 16

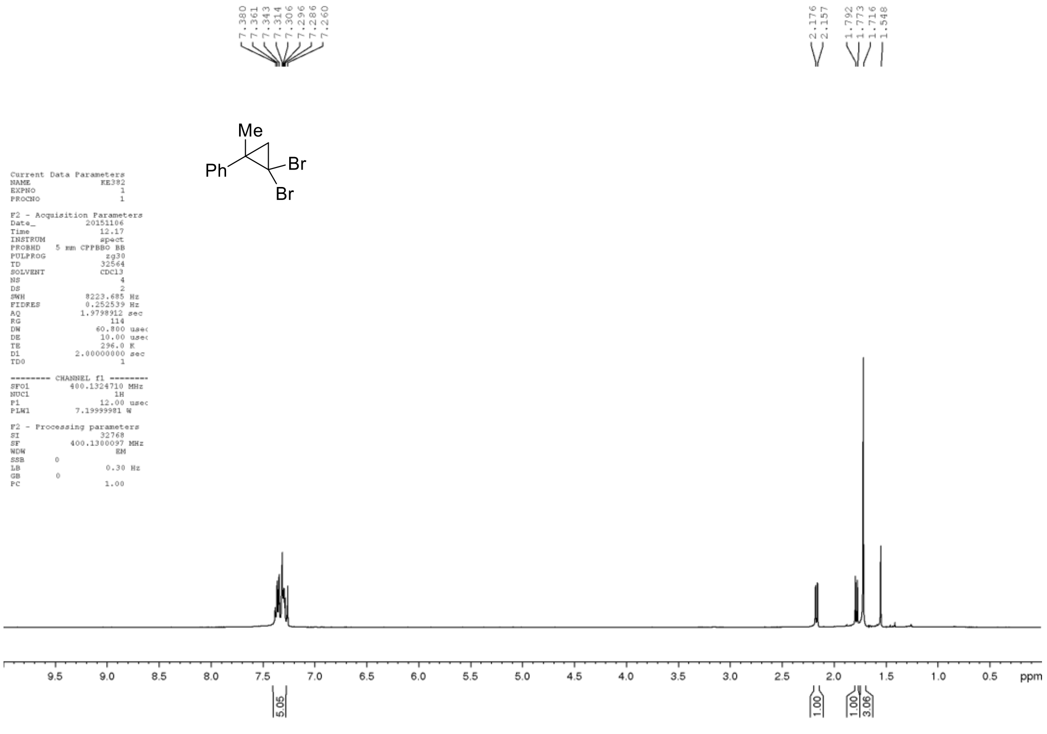

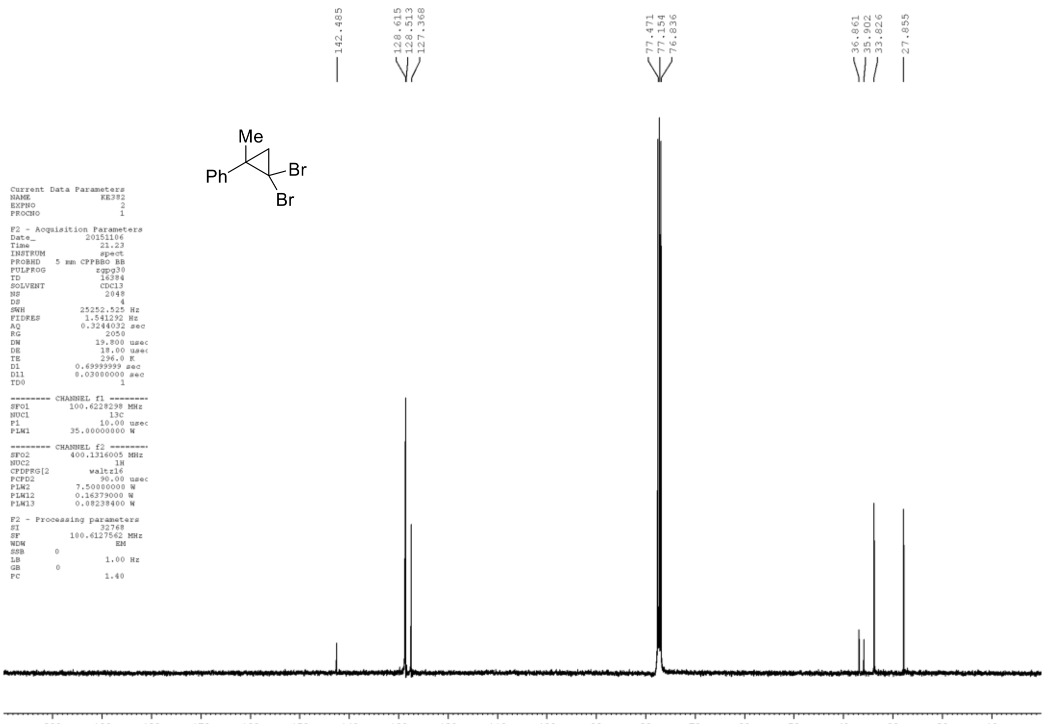

^{1}H-NMR and ^{13}C-NMR 15

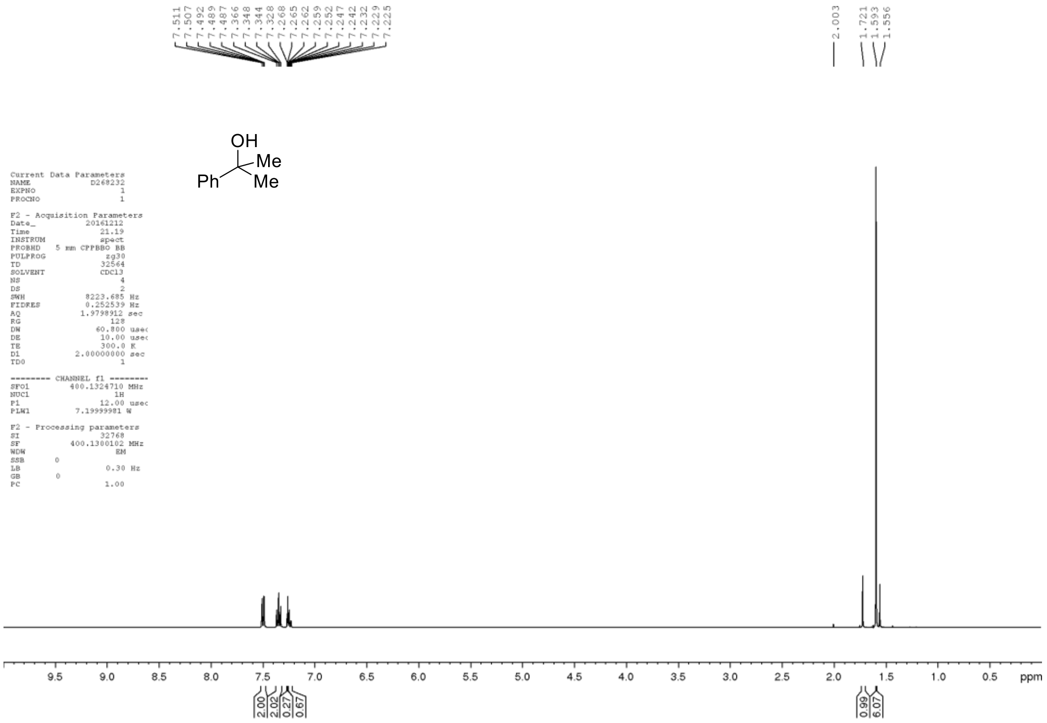

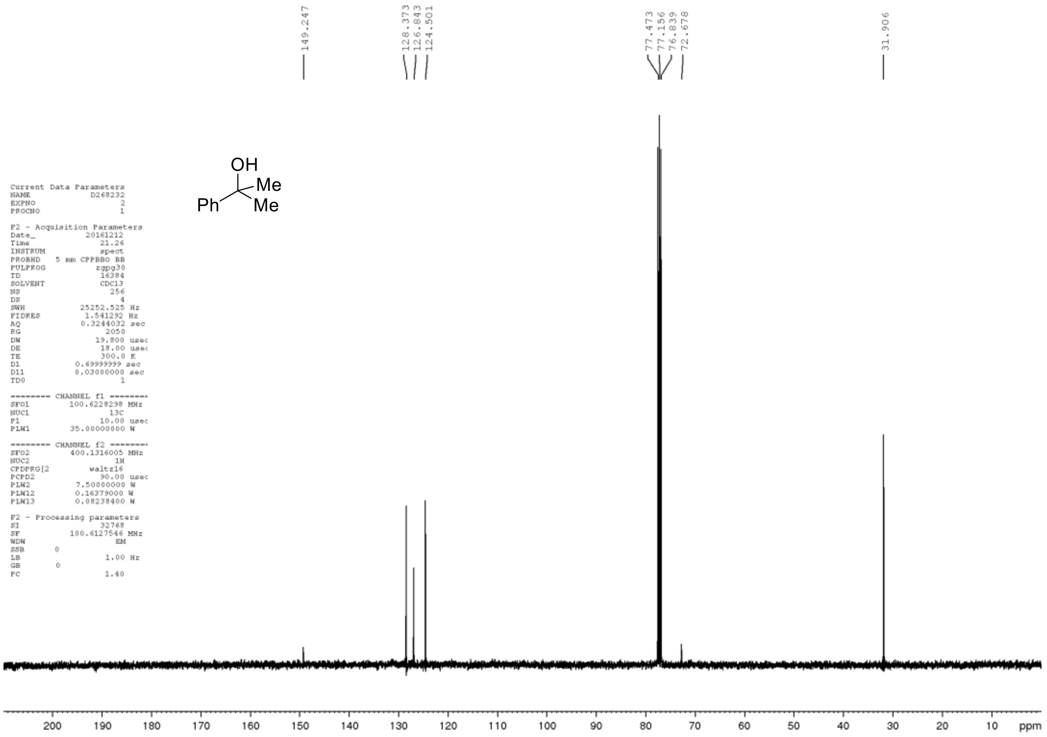

^{1}H-NMR and ^{13}C-NMR 18

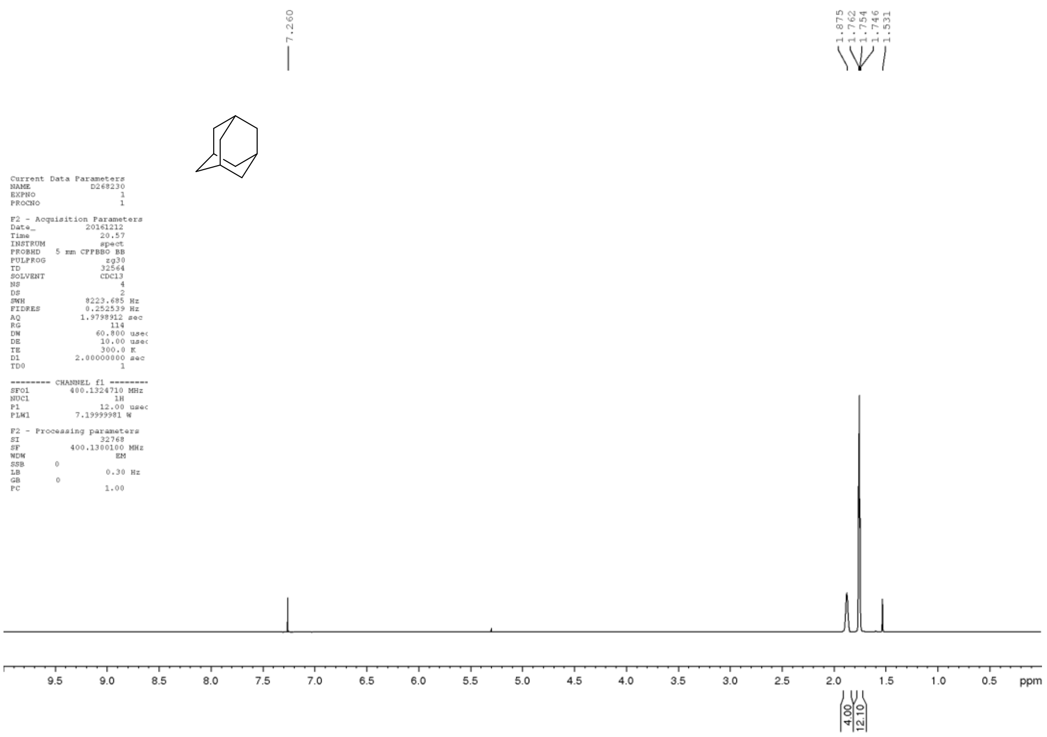

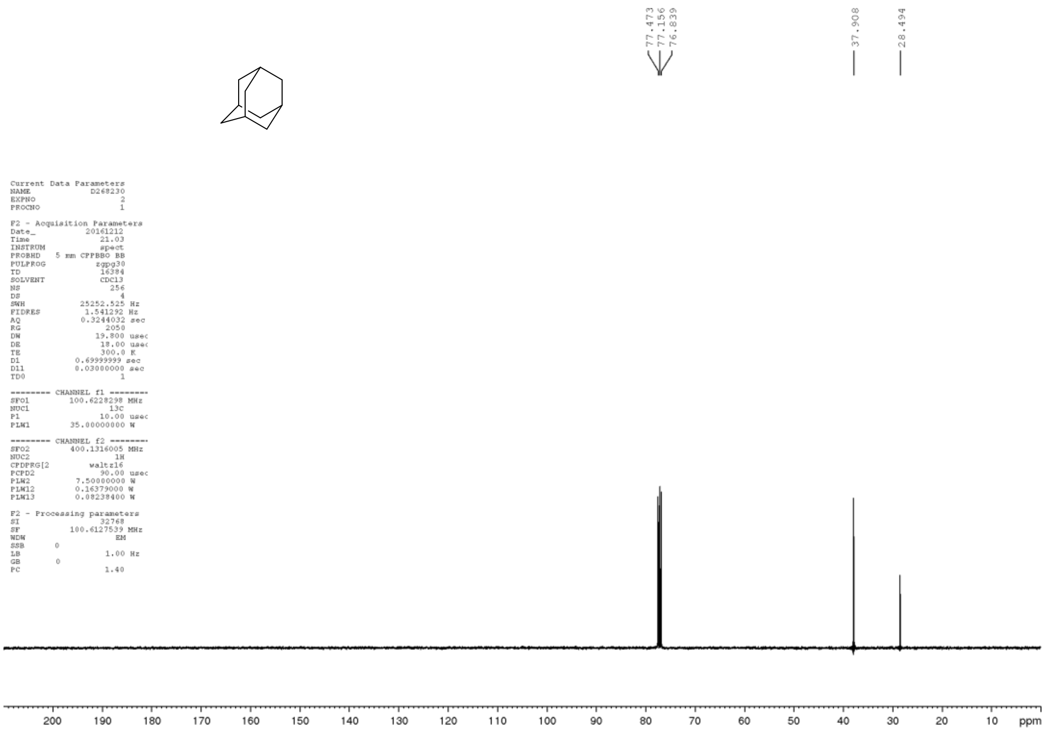

^{1}H-NMR and ^{13}C-NMR 19

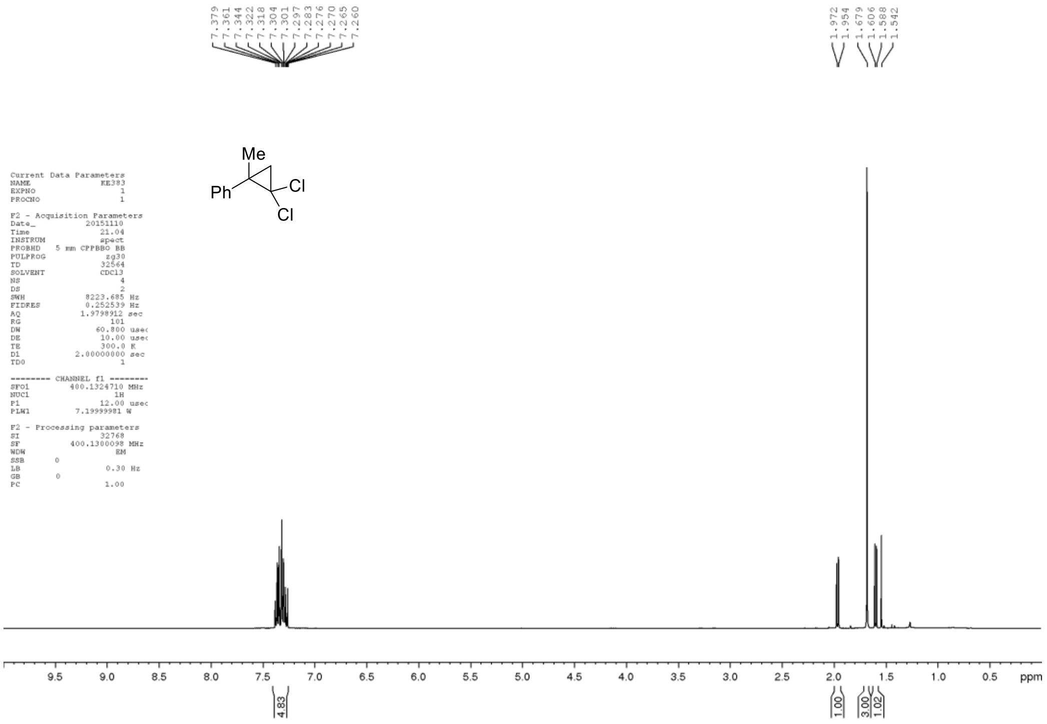

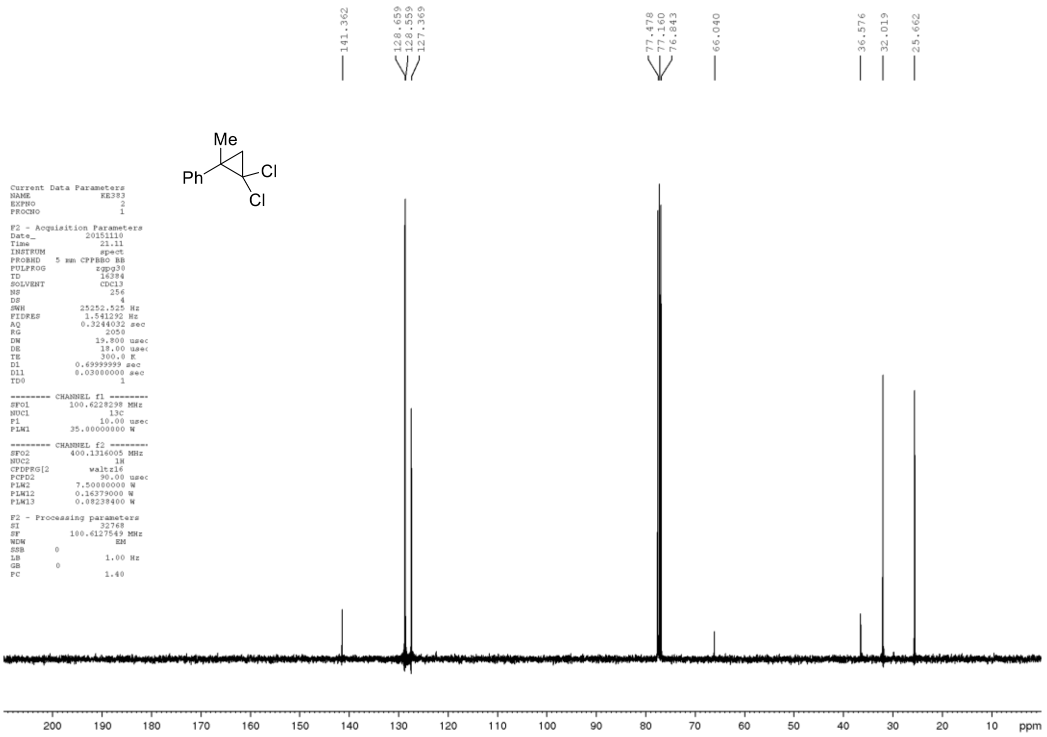

^{1}H-NMR and ^{13}C-NMR 20

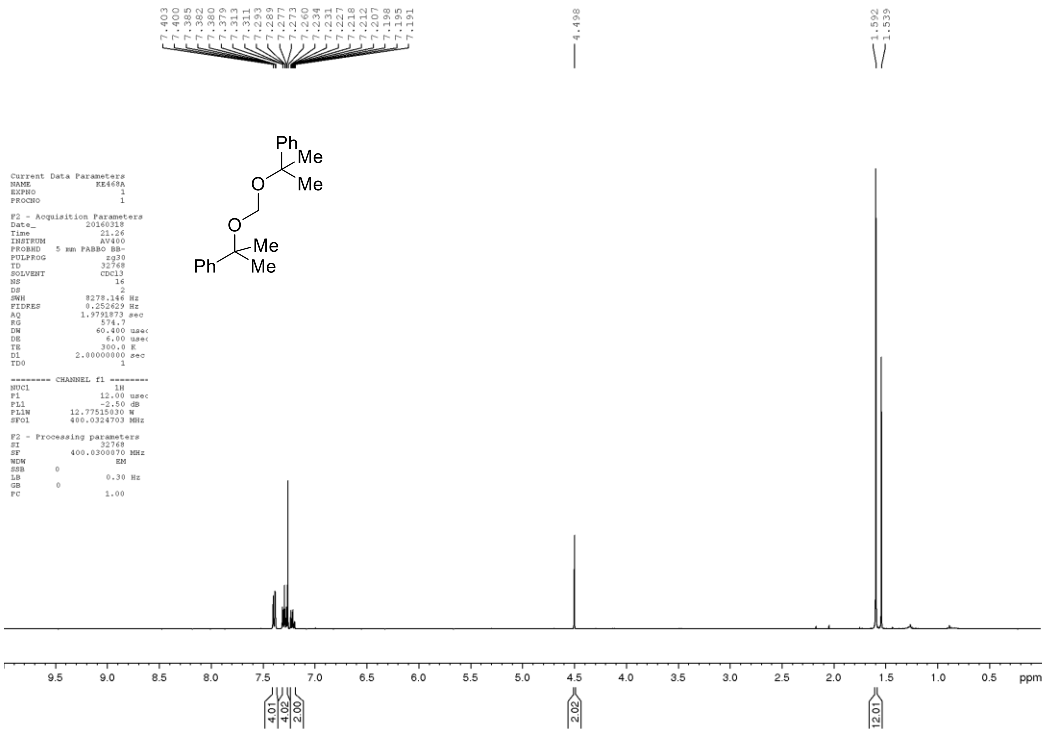

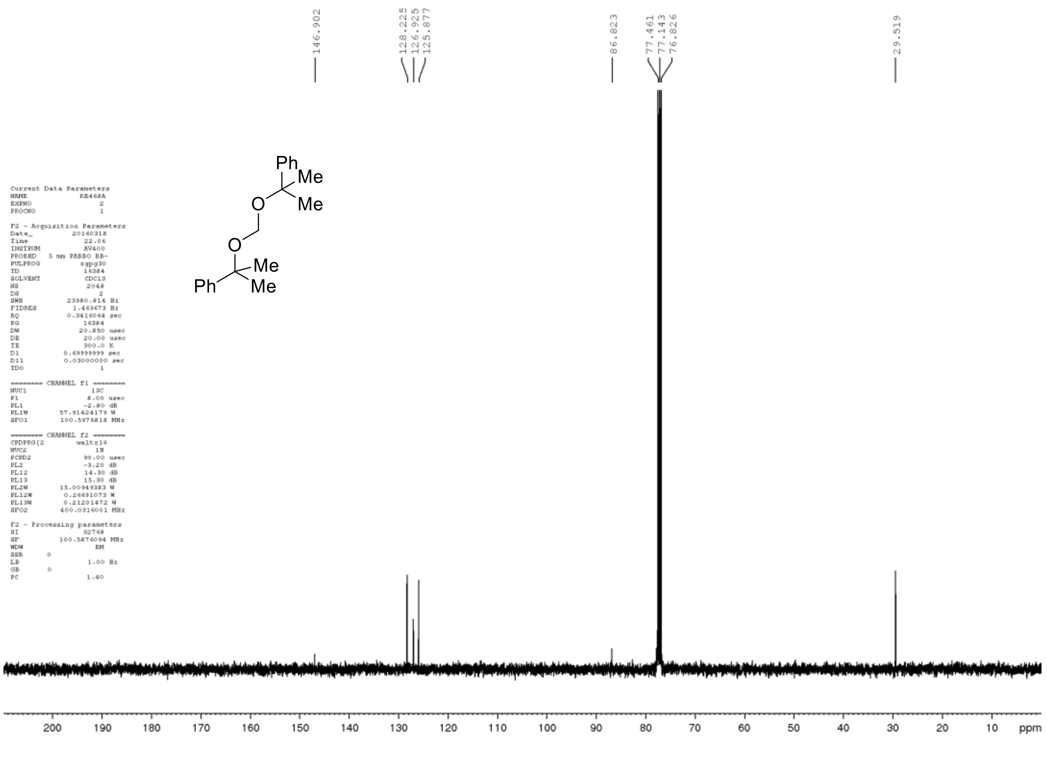

^{1}H-NMR and ^{13}C-NMR 30

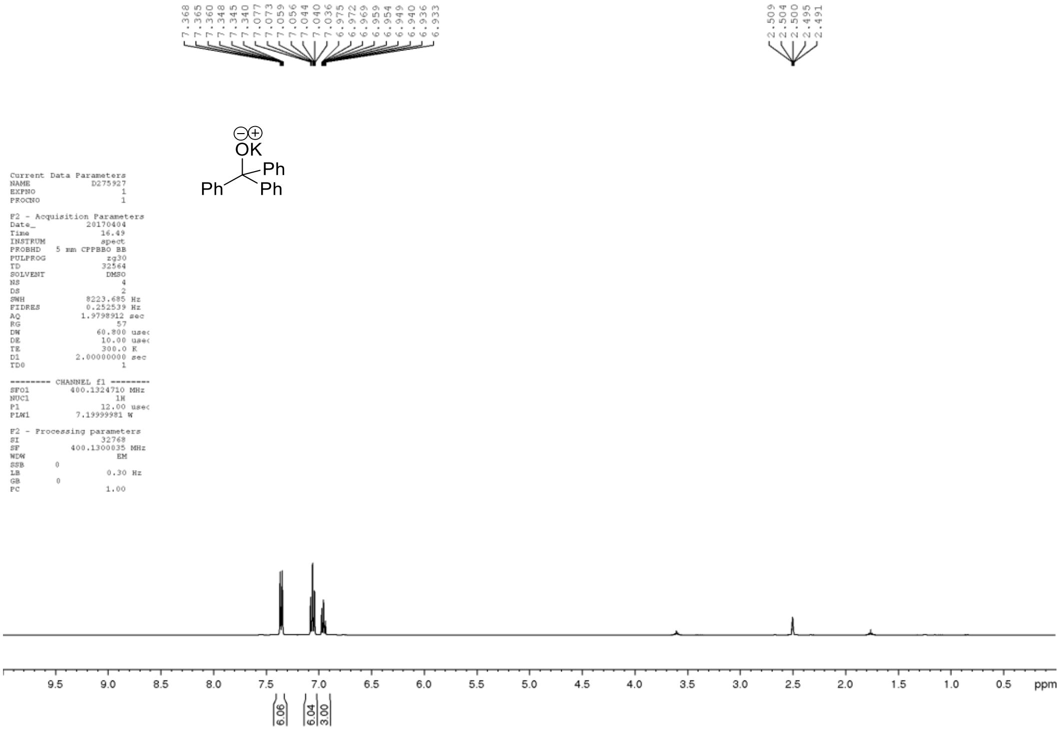

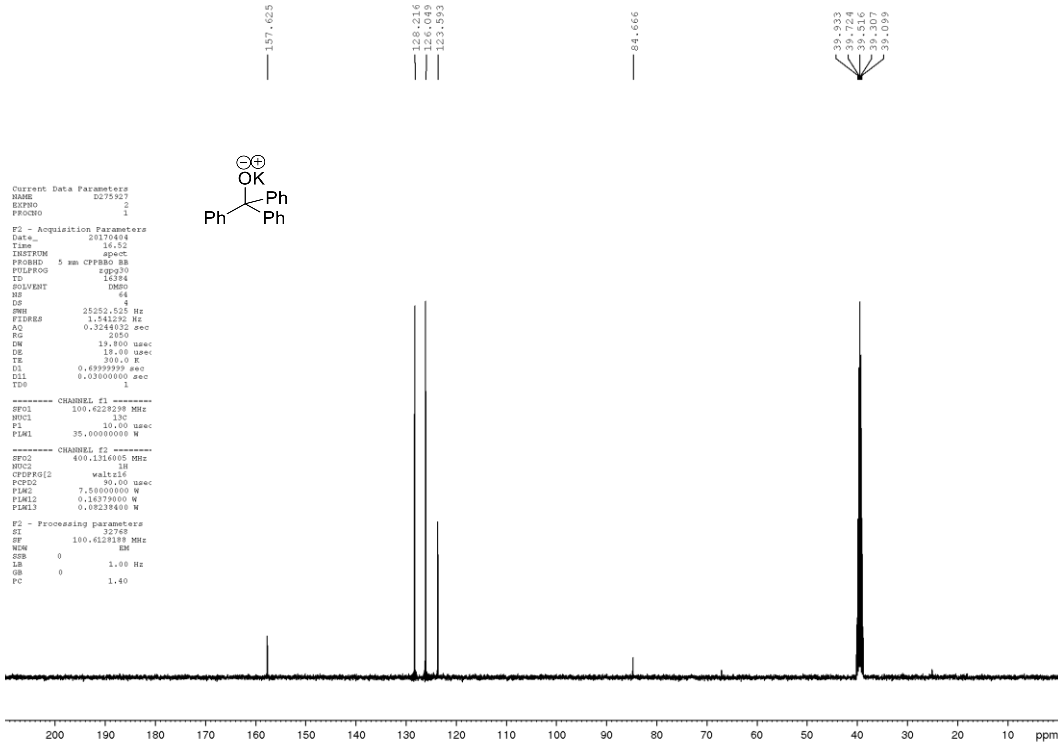

^{1}H-NMR and ^{13}C-NMR 33

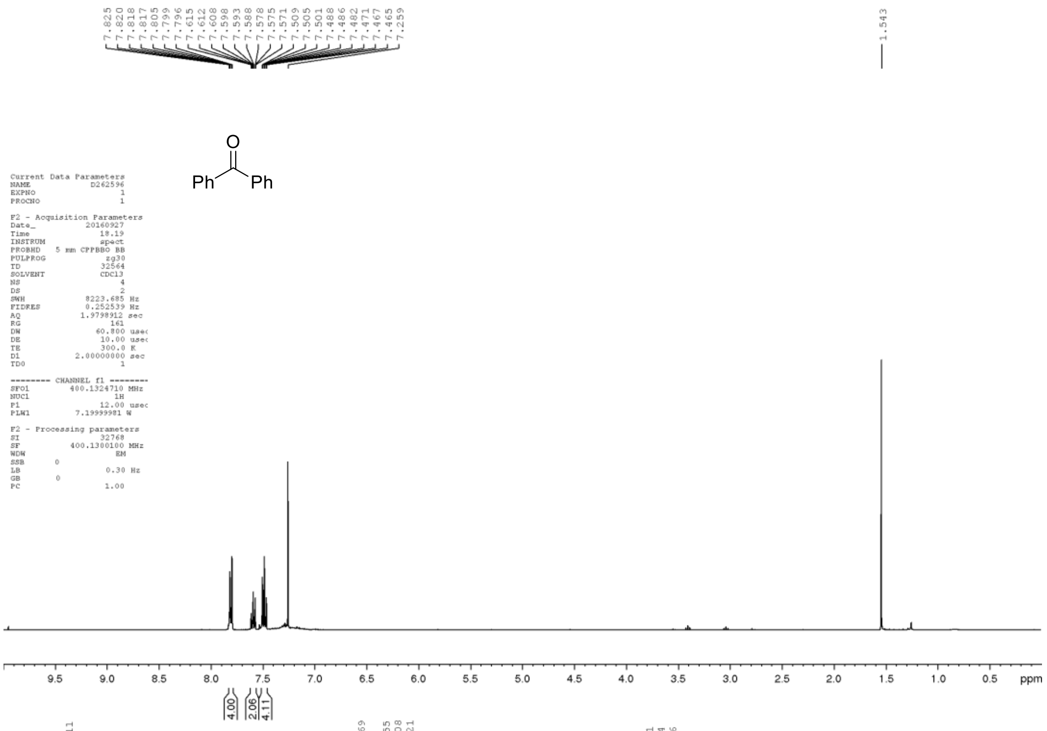
7.825
7.820
7.818
7.817
7.805
7.799
7.796
7.615
7.612
7.608
7.598
7.593
7.588
7.578
7.575
7.571
7.509
7.505
7.501
7.488
7.486
7.482
7.471
7.467
7.465
7.259
1.543
O
Ph
Ph
4.00
2.06
4.11
ppm

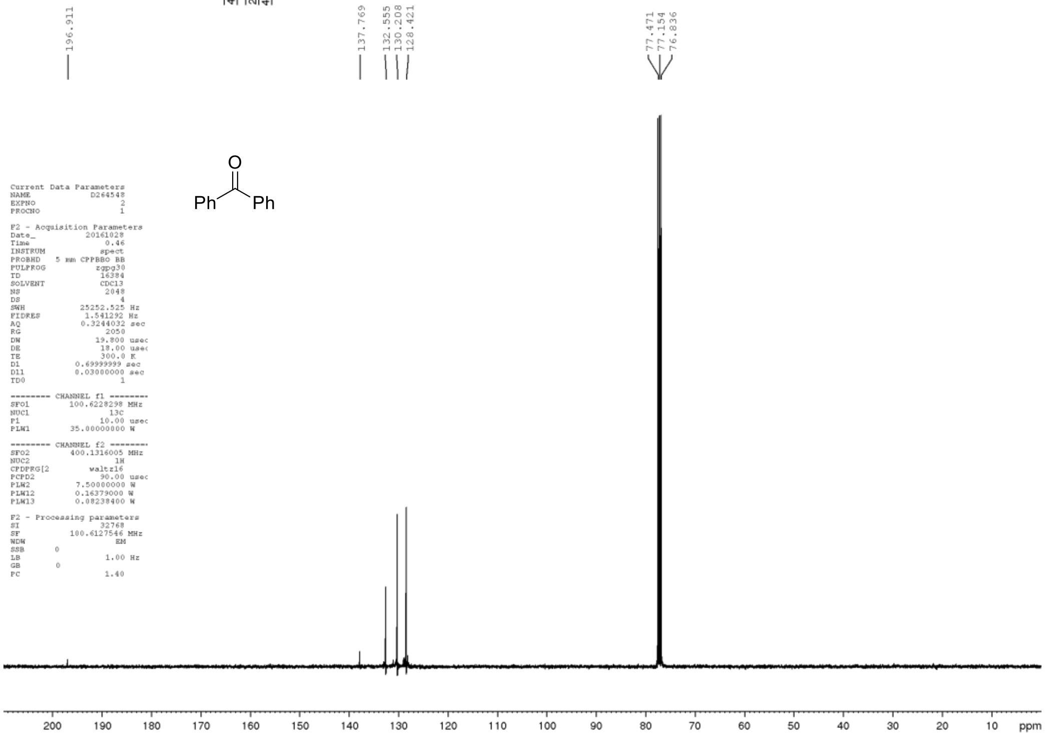
196.911
137.769
132.555
130.208
128.421
77.471
77.154
76.836
O
Ph
Ph
ppm

^{1}H-NMR and ^{13}C-NMR 34

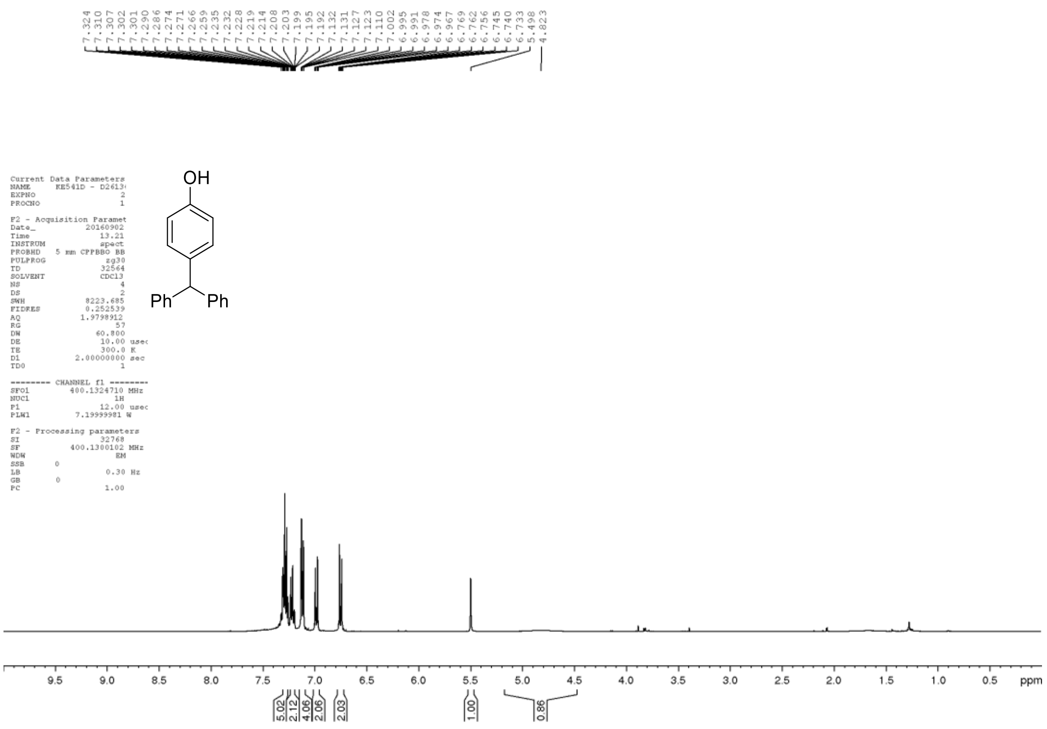

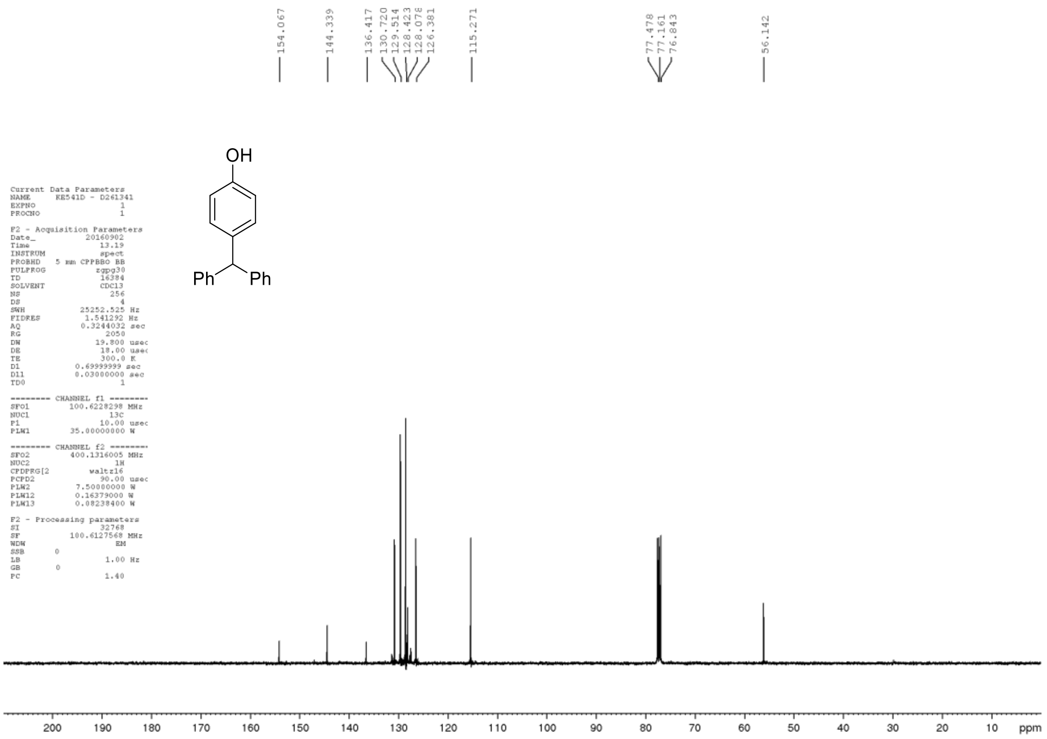

^{1}H-NMR and ^{13}C-NMR 32

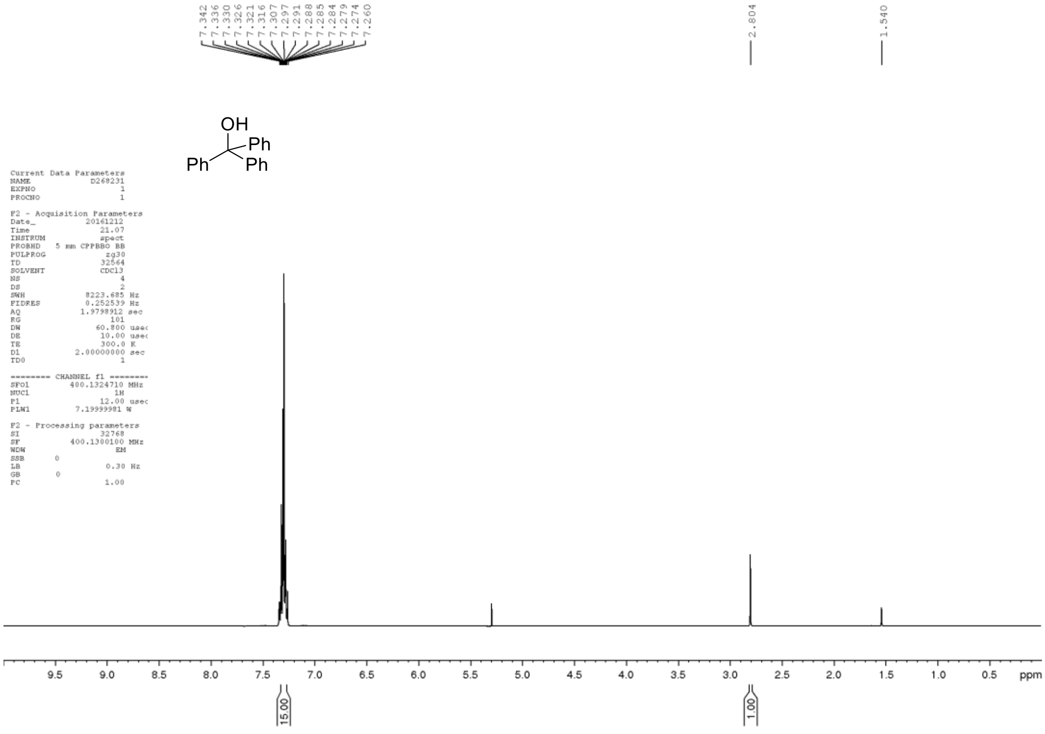

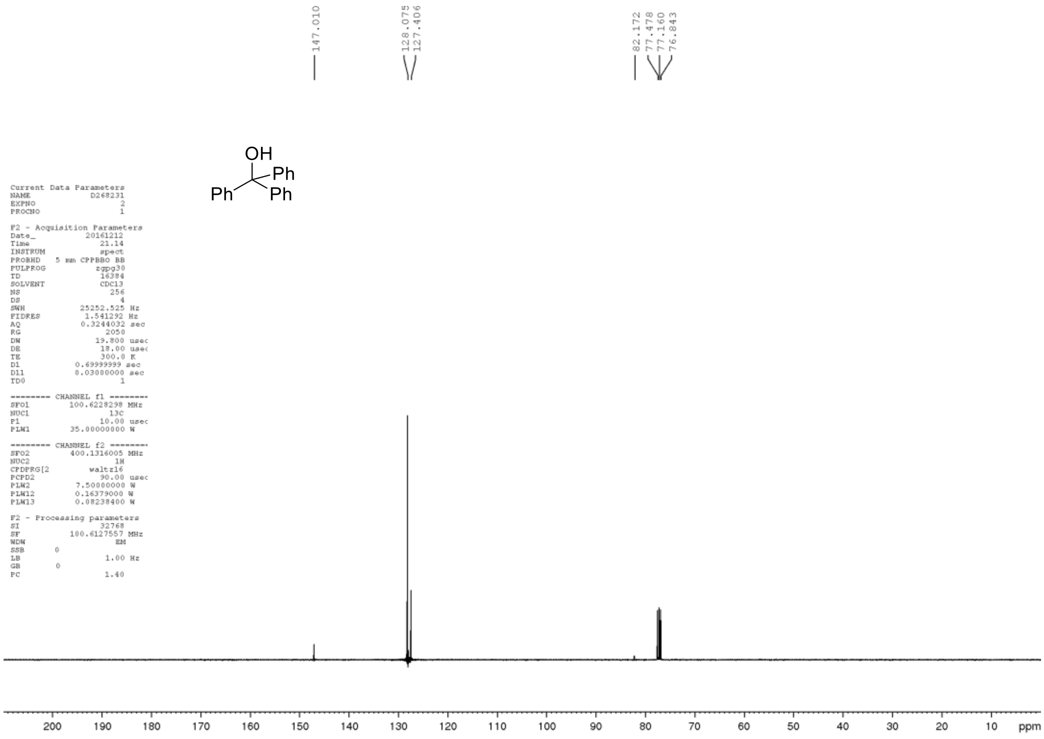

^{1}H-NMR and ^{13}C-NMR 46

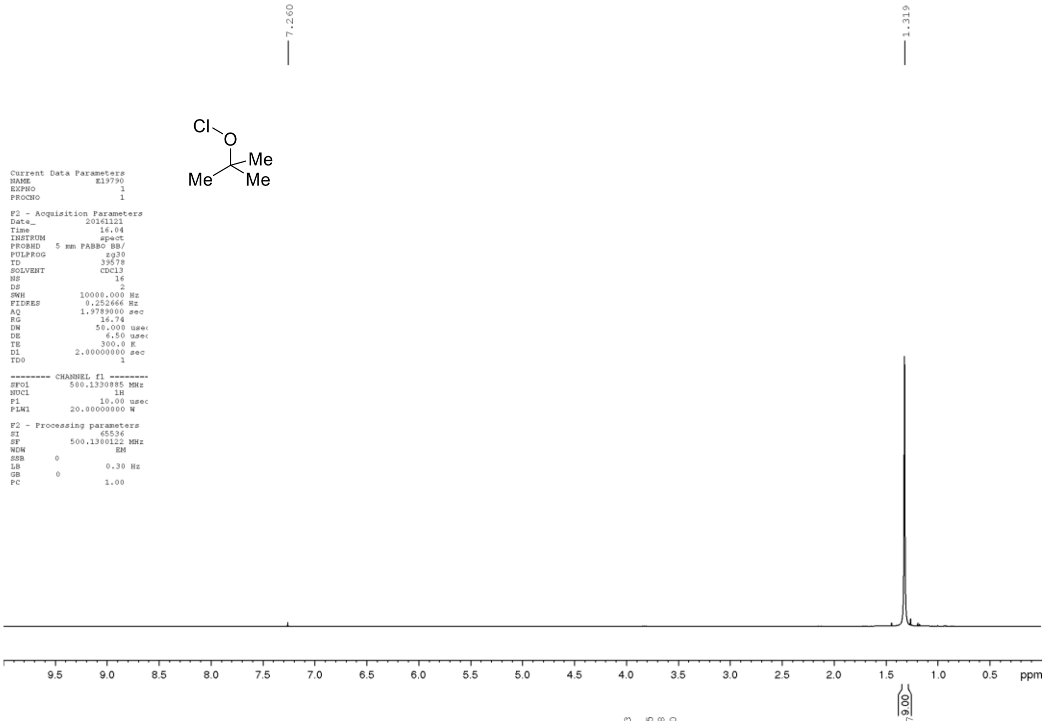

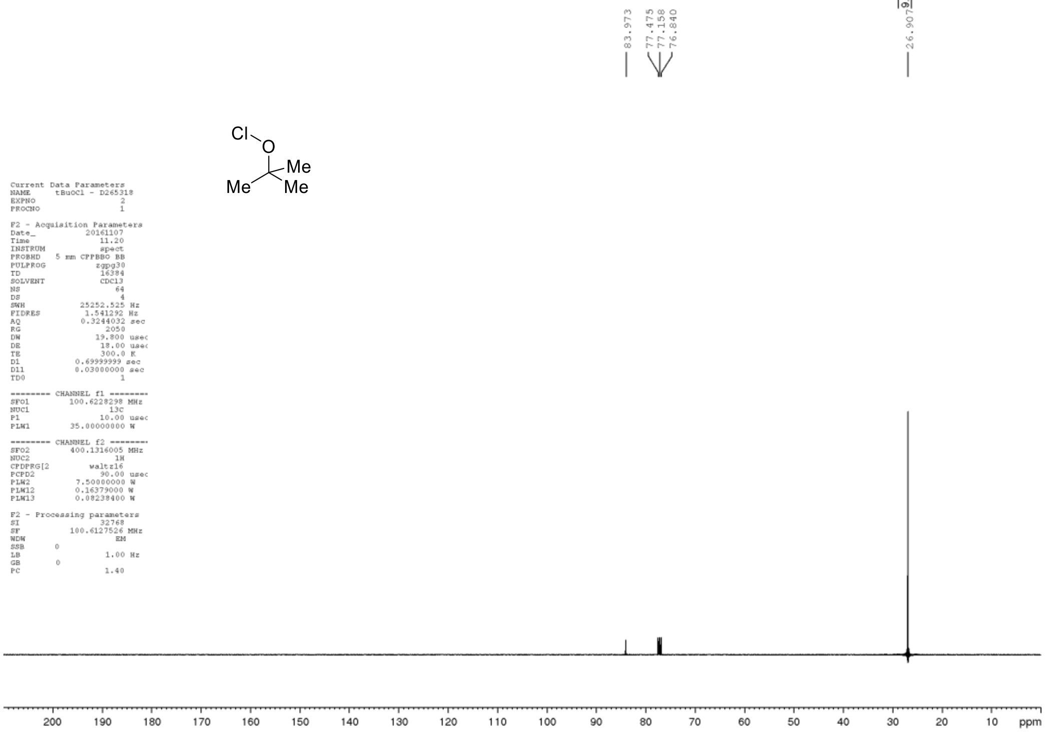

^{1}H-NMR and ^{13}C-NMR *tert*-butyl hypobromite 50

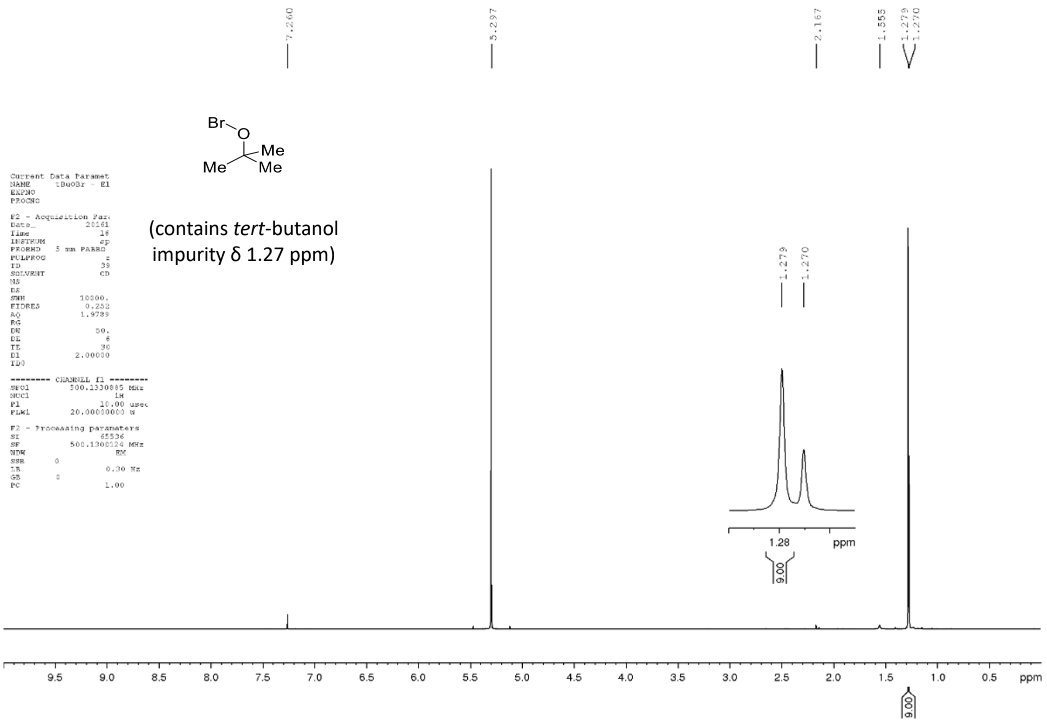

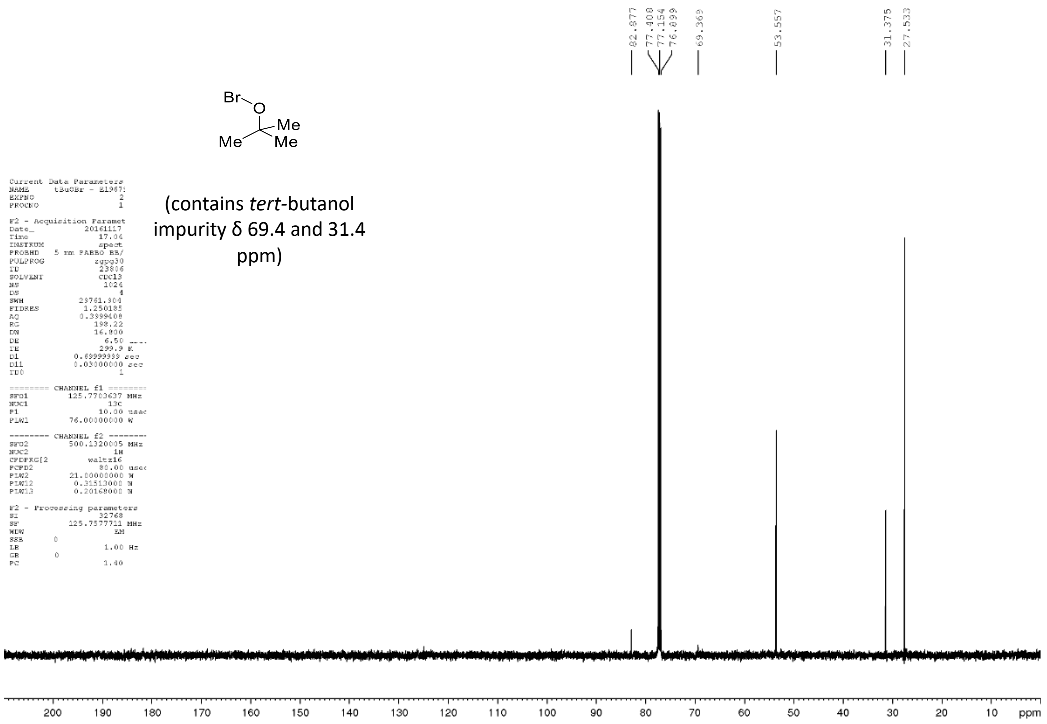

1.5 XYZ coordinates for computational optimisation

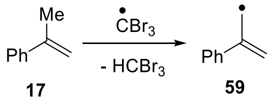

H atom abstraction in 17 /CBr3 radical – starting species 17 and CBr3 radical in benzene

23

-426.9051125

C	-3.90491	-0.96580	-0.22621
C	-3.75423	0.22028	-0.93490
C	-2.81466	1.16321	-0.53024
C	-2.01249	0.94800	0.59521
C	-2.18151	-0.24924	1.30281
C	-3.11299	-1.19521	0.89682
H	-4.62615	-1.70798	-0.54756
H	-4.36334	0.41279	-1.81055
H	-2.70514	2.07400	-1.10586
H	-1.55768	-0.45941	2.16292
H	-3.21328	-2.12069	1.45215
C	-0.98211	1.94772	0.98847
C	-0.47340	2.00075	2.22312
H	-0.80937	1.34756	3.01932
H	0.29386	2.72463	2.47338
C	-0.53525	2.92369	-0.07065
H	-0.28316	2.40786	-1.00190
H	-1.32946	3.64039	-0.29914
H	0.33765	3.48289	0.26573
C	0.98108	-0.25076	-0.12691
Br	0.07561	-0.85567	-1.68954
Br	1.21401	-1.53394	1.26330
Br	2.45321	0.93081	-0.41129

H atom abstraction from 17/CBr3 radical – transition state in benzene

23

-426.8834349

C	4.17606	-1.13666	-0.53288
C	3.82027	-1.28025	0.80551
C	2.92644	-0.39085	1.38987
C	2.38639	0.66507	0.65015
C	2.75495	0.80453	-0.69065
C	3.64219	-0.09082	-1.27887
H	4.86504	-1.83688	-0.99040
H	4.23815	-2.08797	1.39485
H	2.65968	-0.51158	2.43379
H	2.31817	1.60298	-1.27986
H	3.90778	0.02362	-2.32324
C	1.40737	1.60667	1.26104
C	1.48234	2.93075	1.04236
H	2.29363	3.36218	0.46779
H	0.73944	3.60649	1.45056
C	0.29768	1.01752	2.02100
H	0.53096	0.13828	2.62011
H	-0.34978	1.72754	2.53226
H	-0.44283	0.49086	1.12809
C	-1.17109	-0.08734	0.05548
Br	-0.29117	-1.76801	-0.34876
Br	-1.03658	1.20776	-1.37934
Br	-2.98928	-0.31824	0.70604

H atom abstraction from 17 – products 59 and HCBr3 in benzene

23

-426.9178080

C	-3.43817	-1.67132	-0.27794
C	-3.64023	-0.56709	-1.10143
C	-3.10845	0.66920	-0.75541
C	-2.37730	0.82307	0.42482
C	-2.19771	-0.28572	1.25574
C	-2.71640	-1.52726	0.90250
H	-3.84107	-2.63822	-0.55513
H	-4.20963	-0.66968	-2.01787
H	-3.26482	1.52608	-1.40150
H	-1.62590	-0.18269	2.17282
H	-2.54776	-2.38216	1.54689
C	-1.75329	2.13504	0.76979
C	-1.82435	2.60181	2.07395
H	-2.37191	2.06320	2.83593
H	-1.34882	3.53447	2.35094
C	-1.08500	2.83989	-0.22445
H	-1.00182	2.45018	-1.23132
H	-0.62342	3.79539	-0.00779
C	1.01894	-0.02651	0.04643
Br	0.18676	-0.65225	-1.59916
Br	1.41858	-1.50066	1.26207
Br	2.60186	1.06525	-0.31788
H	0.31481	0.62475	0.54665

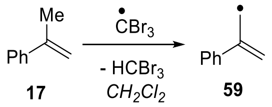

H atom abstraction from 17 – starting species 17 and CBr3 radical in dichloromethane

23

-426.9068042

C	-0.59175	3.06143	0.44122
C	-1.02673	2.66498	-0.82260
C	-1.80766	1.52723	-0.96838
C	-2.18557	0.76412	0.14608
C	-1.73160	1.16674	1.40583

C	-0.94354	2.30540	1.55306
H	0.03010	3.94199	0.55238
H	-0.73792	3.23400	-1.69904
H	-2.10703	1.20768	-1.96006
H	-1.99174	0.58985	2.28556
H	-0.60309	2.59794	2.53975
C	-3.03071	-0.45118	-0.00387
C	-2.86318	-1.54062	1.02446
C	-3.91791	-0.56892	-0.99594
H	-4.08513	0.22324	-1.71640
H	-4.51586	-1.46719	-1.10062
C	0.89088	-0.29981	-0.06697
Br	1.20544	-0.97081	1.69270
Br	2.20482	0.90390	-0.74481
Br	0.18166	-1.55165	-1.32107
H	-3.41533	-2.43363	0.73161
H	-3.23589	-1.21965	2.00120
H	-1.80850	-1.80068	1.14977

H atom abstraction from 17 – transition state in dichloromethane

23

-426.8841066

C	-4.93425	-0.93960	0.31081
C	-4.45968	-1.10433	-0.98683
C	-3.35036	-0.38621	-1.42252
C	-2.70425	0.51624	-0.57224
C	-3.18871	0.67134	0.73140
C	-4.29340	-0.04975	1.16937
H	-5.79446	-1.50352	0.65184
H	-4.95357	-1.79168	-1.66365
H	-3.00198	-0.51388	-2.44109
H	-2.67823	1.34348	1.41216
H	-4.64978	0.07666	2.18501
C	-1.53952	1.31267	-1.05076
C	-1.39905	2.60647	-0.70624
H	-2.13987	3.11551	-0.10122
H	-0.54483	3.18284	-1.04387
C	-0.51527	0.64293	-1.86737
H	-0.82085	-0.25558	-2.39869
H	0.10015	1.30802	-2.47133
H	0.32769	0.19521	-1.04441
C	1.31070	-0.12675	-0.04838
Br	0.28971	-0.44313	1.56750
Br	2.43186	1.45424	0.06253
Br	2.26696	-1.69725	-0.67083

H atom abstraction of 17 CBr_3 radical – products X and $HCBr_3$ in dichloromethane

23

-426.9183180

C	4.11587	0.97577	-0.96558
C	4.15326	-0.41515	-1.02216
C	3.34429	-1.17143	-0.18125
C	2.48362	-0.55089	0.72891
C	2.46284	0.84529	0.78609
C	3.26953	1.60346	-0.05627
H	4.74458	1.56573	-1.62210
H	4.81842	-0.91153	-1.71908
H	3.39178	-2.25371	-0.22384
H	1.78713	1.34464	1.47062
H	3.22978	2.68531	-0.00560
C	1.59060	-1.36238	1.60902
C	1.28807	-0.91016	2.88641
H	1.72996	-0.00937	3.29075
H	0.61904	-1.47671	3.52222
C	1.05011	-2.55022	1.12671
H	1.23036	-2.88931	0.11532
H	0.41636	-3.15755	1.76138
C	-1.19716	-0.04895	0.03483
Br	-0.29426	-0.28200	-1.67931
Br	-1.15922	1.80871	0.63442
Br	-3.02314	-0.74819	-0.01312
H	-0.65202	-0.63812	0.76026

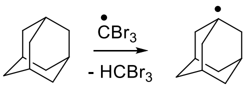

18 56

H atom abstraction from 18 – starting species 18 in dichloromethane

26

-390.6458290

C	-1.10154	0.02223	-1.38950
H	-0.87252	-0.32352	-2.40376
H	-2.13602	0.38348	-1.39153
C	-0.14894	1.16304	-0.99714
H	-0.25448	1.98966	-1.70645
C	-0.95480	-1.14017	-0.39527
H	-1.63379	-1.95113	-0.67608
C	0.49445	-1.65081	-0.41808
H	0.74433	-2.02000	-1.41905
H	0.60526	-2.48969	0.27824
C	1.29871	0.64722	-1.01915
H	1.98763	1.45755	-0.75565
H	1.55958	0.30977	-2.02852
C	1.45112	-0.51376	-0.02418
H	2.48252	-0.87897	-0.04101
C	1.10161	-0.02221	1.38921
H	1.22124	-0.83941	2.10939
H	1.78726	0.77959	1.68541
C	-0.49477	1.65065	0.41869
H	-1.52101	2.03405	0.44120
H	0.17003	2.47435	0.70211
C	-1.29900	-0.64750	1.01887
H	-2.33607	-0.29479	1.04870
H	-1.21276	-1.47342	1.73385
C	-0.34658	0.49150	1.41647
H	-0.59276	0.84140	2.42368

H atom abstraction from 18 – starting species CBr_3 radical in dichloromethane

4

-78.0020647

C	0.00044	-0.00044	0.33966
Br	1.85239	-0.26784	-0.01942
Br	-1.15836	-1.46920	-0.01941
Br	-0.69410	1.73712	-0.01940

H atom abstraction of 18 – transition state in dichloromethane

30

-468.6412626

C	-1.69951	0.40968	-1.37350
H	-1.35970	-0.26981	-2.16298
H	-1.26297	1.39556	-1.56598
C	-1.27870	-0.11942	-0.01398
H	-0.01580	-0.12510	-0.00522
C	-3.24812	0.48677	-1.37206
H	-3.58923	0.88147	-2.33389
C	-3.81937	-0.91892	-1.13979
H	-3.51459	-1.58326	-1.95483
H	-4.91350	-0.87447	-1.13991
C	-1.77575	-1.53624	0.19124
H	-1.39852	-1.96322	1.12699
H	-1.45054	-2.17692	-0.63501
C	-3.32356	-1.47091	0.21180
H	-3.72308	-2.47631	0.36899
C	-3.79325	-0.53614	1.33668
H	-4.88798	-0.50047	1.33763
H	-3.47812	-0.92654	2.31069
C	-1.68675	0.80869	1.11071
H	-1.28831	1.81234	0.92686
H	-1.29891	0.46045	2.07465
C	-3.71027	1.41610	-0.24248
H	-3.32890	2.43104	-0.39422
H	-4.80407	1.47528	-0.24405
C	-3.23091	0.87094	1.11601
H	-3.55531	1.52897	1.92712
C	1.37627	-0.02003	-0.01850
Br	1.72052	1.87164	-0.36574
Br	2.14090	-1.19722	-1.37614
Br	1.91769	-0.54391	1.78549

H atom abstraction from 18 – product 56 in dichloromethane

25

-389.9798509

C	-0.90747	-1.12797	1.07358
H	-1.92515	-0.97635	1.44813
H	-0.54442	-2.09287	1.44230
C	0.00178	-0.00498	1.48362
C	-0.91612	-1.13082	-0.48126
H	-1.56639	-1.93233	-0.84494
C	-1.43462	0.22863	-0.97862
H	-2.45908	0.39005	-0.62550
H	-1.46100	0.23602	-2.07377
C	-0.51662	1.34640	1.08041
H	0.12724	2.14995	1.45289
H	-1.53112	1.51522	1.45616
C	-0.52385	1.36006	-0.47432
H	-0.89524	2.32476	-0.83336
C	0.90989	1.13046	-0.98045
H	0.92292	1.14984	-2.07578
H	1.56426	1.93703	-0.63197
C	1.42982	-0.22903	1.07371
H	1.80724	-1.18921	1.44059
H	2.08563	0.56422	1.44743
C	0.51992	-1.34927	-0.98734
H	0.89383	-2.31972	-0.64288
H	0.52654	-1.36656	-2.08268
C	1.43704	-0.22444	-0.48103
H	2.45613	-0.38419	-0.84639

H atom abstraction from 18 – product $HCBr_3$ in dichloromethane

5

-78.6661292

C	0.00056	-0.00040	0.53201
Br	1.75864	-0.62524	-0.04580
Br	-1.42110	-1.20979	-0.04575
Br	-0.33767	1.83511	-0.04573
H	0.00105	-0.00068	1.61262

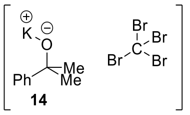

Neutral complex of 14 and CBr_4 in dichloromethane

27

-1116.1116760

C	3.10600	0.93009	0.61016
C	2.68297	0.81112	2.08946
H	3.50789	0.50312	2.73667
H	2.32364	1.78662	2.42866
H	1.87227	0.08711	2.19661
C	4.31261	1.88956	0.52154
H	4.62136	1.99333	-0.52192
H	4.01315	2.87211	0.89887
H	5.16877	1.53943	1.10545
O	2.07777	1.45022	-0.15433
C	3.56206	-0.44960	0.10293
C	3.03091	-0.98119	-1.07248
C	4.53174	-1.19650	0.78308
C	3.44692	-2.22296	-1.55210
H	2.28262	-0.40565	-1.60253
C	4.95150	-2.43506	0.30863
H	4.97143	-0.80792	1.69593
C	4.40798	-2.95647	-0.86405
H	3.01802	-2.61808	-2.46680
H	5.70410	-2.99399	0.85377
H	4.73220	-3.92206	-1.23477
Br	-0.06633	0.46111	-0.01173

C	-1.95735	-0.26662	0.04902
Br	-3.14756	0.82699	-1.09293
Br	-2.64399	-0.22230	1.90371
Br	-1.99814	-2.13452	-0.59663
K	1.24586	3.63343	-0.93669

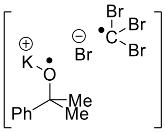

Triplet complex 44 in dichloromethane

27

-1116.0776501

C	2.30193	-1.04484	0.70234
C	1.46279	-1.77821	-0.35183
H	2.01937	-2.61793	-0.77089
H	0.54809	-2.15406	0.11012
H	1.19767	-1.08111	-1.14787
C	2.57293	-1.99402	1.90724
H	3.15012	-1.47761	2.67451
H	1.62566	-2.33948	2.32256
H	3.14293	-2.85097	1.54593
O	1.56608	-0.03774	1.28604
C	3.62712	-0.54028	0.13215
C	3.95004	0.81504	0.16401
C	4.53801	-1.43677	-0.43376
C	5.16100	1.26634	-0.35685
H	3.23946	1.51694	0.58047
C	5.74696	-0.98756	-0.95468
H	4.30882	-2.49665	-0.47177
C	6.06335	0.36839	-0.91725
H	5.39506	2.32445	-0.32957
H	6.44147	-1.69666	-1.38991
H	7.00381	0.72032	-1.32460
Br	0.49425	1.83738	-1.00419
C	-2.22121	-0.32519	-0.17504
Br	-2.02620	-1.20291	1.51587
Br	-2.12953	-1.44307	-1.71261
Br	-3.51162	1.07993	-0.23179
K	0.06526	1.94213	2.09261

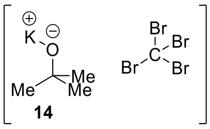

Neutral complex of 14 and CBr_4 in dichloromethane

20

-924.4005342

C	3.68603	-0.95755	-0.00045
C	3.38994	-1.79739	1.25294
H	4.00014	-2.70535	1.28365
H	3.59906	-1.20742	2.15015
H	2.33679	-2.08982	1.27196
C	5.17655	-0.58026	-0.00169
H	5.40793	0.01591	-0.88910

H	5.40934	0.01548	0.88565
H	5.81889	-1.46676	-0.00236
O	2.93691	0.21790	0.00033
C	3.38738	-1.79711	-1.25348
C	-1.41957	-0.05845	-0.00013
K	2.83217	2.66403	0.00262
H	3.99700	-2.70543	-1.28527
H	2.33392	-2.08900	-1.27099
H	3.59542	-1.20717	-2.15097
Br	-2.07517	-1.39505	-1.30191
Br	-2.09604	-0.54006	1.79686
Br	-2.14752	1.71745	-0.49499
Br	0.62939	-0.00450	-0.00076

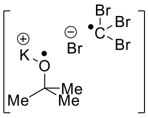

45

Triplet complex 45 in dichloromethane

20

-924.3685022

C	4.70433	-0.31992	0.25852
C	4.59623	-1.79140	0.66061
H	5.48883	-2.33459	0.34233
H	4.49407	-1.87981	1.74398
H	3.71962	-2.23312	0.18318
C	5.90609	0.34368	0.98065
H	5.96968	1.40266	0.72715
H	5.81279	0.23106	2.06114
H	6.81402	-0.15968	0.64270
O	3.62317	0.39507	0.75021
C	4.83439	-0.14945	-1.25482
C	-2.06866	-0.03608	0.11930
K	1.78092	2.04832	-0.29321
H	5.71972	-0.67292	-1.62259
H	3.94853	-0.56239	-1.74140
H	4.92257	0.90922	-1.51111
Br	-3.15680	-1.15085	-0.97859
Br	-2.04671	-0.44498	1.98252
Br	-2.06619	1.82747	-0.32393
Br	1.05408	-0.94765	-0.84691

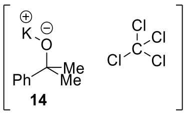

Neutral complex of 14 and CCl_4 in carbon tetrachloride

27

-2903.5754123

C	2.29240	1.12884	0.64758
C	1.95731	0.99349	2.15010
H	2.78116	0.58595	2.74215
H	1.71297	1.98667	2.53662
H	1.08514	0.34798	2.27901
C	3.56417	1.99612	0.50483

H	3.80938	2.10474	-0.55511
H	3.36302	2.98780	0.92066
H	4.42902	1.56777	1.02040
O	1.24533	1.71555	-0.01778
C	2.62482	-0.26963	0.08274
C	2.00048	-0.72218	-1.07906
C	3.56668	-1.11112	0.68725
C	2.29093	-1.97510	-1.61611
H	1.27553	-0.07034	-1.54943
C	3.86206	-2.36313	0.15710
H	4.08170	-0.78798	1.58596
C	3.22166	-2.80411	-0.99896
H	1.78737	-2.30502	-2.51865
H	4.59301	-2.99670	0.64743
H	3.44763	-3.78058	-1.41161
C	-2.55237	-0.56021	0.18435
K	-0.27510	3.08460	-1.15816
Cl	-3.75660	0.46765	-0.64398
Cl	-0.98260	0.27978	0.21369
Cl	-3.10512	-0.88079	1.84009
Cl	-2.39511	-2.09310	-0.69620

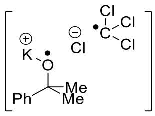

60

Triplet complex of 60 in carbon tetrachloride

27

-2903.5364951

C	-1.80480	1.60632	-0.46437
C	-2.30812	1.27837	-1.87784
H	-3.35447	0.97347	-1.85532
H	-2.21206	2.16235	-2.50998
H	-1.71792	0.46443	-2.30189
C	-2.66274	2.76194	0.14295
H	-2.28228	3.03372	1.12724
H	-2.65176	3.62725	-0.51988
H	-3.68247	2.38795	0.24419
O	-0.55857	2.18283	-0.53988
C	-1.83634	0.40052	0.47379
C	-0.72678	0.06598	1.24467
C	-2.98897	-0.38387	0.57314
C	-0.75446	-1.04480	2.08541
H	0.18814	0.64186	1.19501
C	-3.02018	-1.48943	1.41537
H	-3.87150	-0.13999	-0.00853
C	-1.89894	-1.82777	2.17097
H	0.13462	-1.28744	2.65710
H	-3.92019	-2.08997	1.47852
H	-1.92202	-2.69508	2.82015
C	0.82704	-1.51652	-0.84676
K	2.02323	2.65420	-0.00182
Cl	2.79126	0.59266	1.80325
Cl	1.19342	-0.13550	-1.82774
Cl	2.15241	-2.43336	-0.29053
Cl	-0.58621	-2.38118	-1.30128

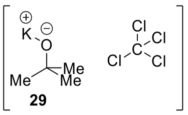

Neutral complex of 29 and CCl_4 in carbon tetrachloride

20

-2711.8636814

C	-3.02634	-0.76219	0.00198
C	-2.69791	-1.61317	-1.24061
H	-3.28973	-2.53383	-1.28056
H	-2.89900	-1.03050	-2.14476
H	-1.63798	-1.88314	-1.23742
C	-4.52907	-0.42322	-0.02676
H	-4.78689	0.17543	0.85221
H	-4.75553	0.16477	-0.92150
H	-5.15837	-1.31976	-0.03256
O	-2.27628	0.39675	0.01073
C	-2.74169	-1.60166	1.26262
C	2.09086	-0.20150	0.00142
K	-1.49979	2.60659	-0.00004
Cl	2.78925	1.37273	-0.46841
Cl	0.31288	-0.07798	0.02300
Cl	2.59502	-1.43469	-1.17262
Cl	2.69393	-0.63997	1.61483
H	-3.33004	-2.52506	1.28827
H	-1.68088	-1.86538	1.29983
H	-2.97875	-1.01277	2.15387

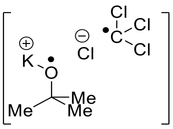

61

Triplet complex 61 in carbon tetrachloride 61

20

-2711.8260835

C	-2.83443	-0.68658	-0.00449
C	-2.28839	-1.77393	-0.93147
H	-2.61761	-2.75876	-0.59371
H	-2.65334	-1.61892	-1.94951
H	-1.19800	-1.74960	-0.93819
C	-4.36360	-0.70193	0.07124
H	-4.71590	0.08841	0.73563
H	-4.79215	-0.54819	-0.92155
H	-4.70979	-1.66542	0.45062
O	-2.38719	0.55854	-0.39227
C	-2.22821	-0.84550	1.42033
C	1.95988	-0.55890	-0.13473
K	-0.64093	2.50685	-0.67527
Cl	0.56444	1.52425	1.71236
Cl	1.23764	-0.16704	-1.66020
Cl	3.60471	-0.12491	0.05089
Cl	1.46236	-2.04237	0.56768
H	-2.51259	-1.83854	1.77564
H	-1.14322	-0.74953	1.39094

H	-2.62952	-0.08559	2.09070
H	-1.45529	0.42553	0.48601

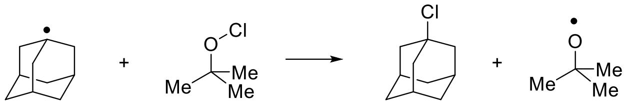

54 46 47 62

Cl abstraction from 46 – starting species 54 and 46 in dichloromethane

25
-389.9787578

C	1.25958	-1.21146	0.30720
H	2.15841	-1.04459	0.91215
H	1.28647	-2.24614	-0.04961
C	1.25632	-0.23910	-0.89902
H	2.14286	-0.41326	-1.51406
C	0.00009	-0.97690	1.15805
H	0.00017	-1.66874	2.00589
C	-0.00057	0.46994	1.67516
H	0.88261	0.64453	2.29962
H	-0.88417	0.64393	2.29922
C	1.25596	1.20874	-0.36549
H	1.27791	1.91142	-1.20471
H	2.15506	1.38132	0.23669
C	-0.00056	1.44414	0.48793
H	-0.00097	2.47407	0.85759
C	-1.25657	1.20799	-0.36601
H	-2.15600	1.37998	0.23582
H	-1.27862	1.91067	-1.20523
C	0.00046	-0.47349	-1.68393
H	0.00080	-1.13093	-2.54645
C	-1.25886	-1.21235	0.30669
H	-1.28476	-2.24705	-0.05013
H	-2.15805	-1.04622	0.91131
C	-1.25581	-0.23990	-0.89946
H	-2.14201	-0.41466	-1.51482

Cl abstraction of 46 – products 47 and 62 in dichloromethane

40
-1083.2201766

C	0.94175	-0.08291	-0.69188
H	0.69362	-0.04390	-1.75329
C	1.84639	-1.28100	-0.39802
C	1.60627	1.22181	-0.24969
H	1.33285	-2.20030	-0.69102
C	2.22126	-1.32695	1.09047
C	3.12301	-1.10026	-1.24206
H	0.92436	2.05478	-0.43929
C	1.98202	1.16360	1.23822
C	2.88324	1.38683	-1.09495
H	2.88377	-2.18078	1.26578
H	1.32648	-1.47550	1.70167
C	2.92680	-0.02191	1.48884
H	2.87115	-1.08284	-2.30775
H	3.78515	-1.95571	-1.07511
C	3.82963	0.20358	-0.84352
H	1.08185	1.06805	1.85182
H	2.47286	2.10097	1.51904
H	2.62682	1.44438	-2.15822
H	3.37239	2.32789	-0.82438
H	3.18992	-0.05924	2.54977
C	4.19948	0.15078	0.64661
H	4.73479	0.32603	-1.44510
H	4.71722	1.07208	0.93487
H	4.88589	-0.68295	0.83041
Cl	-0.68541	-0.28519	0.11879
O	-3.61021	-0.08282	1.13598
C	-4.38060	0.04668	-0.00738
C	-5.81978	0.13303	0.56072
H	-5.92173	1.00112	1.21269
H	-6.50483	0.23494	-0.28326
H	-6.06721	-0.77216	1.11603
C	-4.23705	-1.18738	-0.90021
H	-3.20748	-1.27182	-1.25421
H	-4.49292	-2.08870	-0.34001
H	-4.89770	-1.10934	-1.76650
C	-4.03019	1.33601	-0.75350
H	-4.14041	2.19677	-0.09135
H	-2.99778	1.29134	-1.10652
H	-4.68870	1.46423	-1.61544

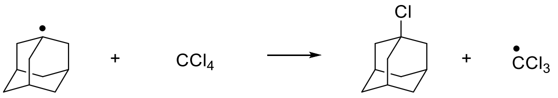

56 47

Cl abstraction from CCl_4 – starting species CCl_4 and 56 in dichloromethane

30
-2268.8442784

C	-1.70633	-0.00075	-1.27199
H	-1.40594	-0.00053	-2.31553
C	-2.24373	1.25671	-0.65558
C	-2.24679	-1.25755	-0.65693
H	-1.79936	2.14176	-1.11831
C	-1.96852	1.25352	0.86154
C	-3.77896	1.26152	-0.87995
H	-1.80459	-2.14320	-1.12059
C	-1.97155	-1.25665	0.86019
C	-3.78204	-1.25842	-0.88131
H	-2.39911	2.15341	1.31423
H	-0.88967	1.27464	1.04467
C	-2.58623	-0.00117	1.49782
H	-3.99726	1.28780	-1.95241
H	-4.21512	2.16047	-0.42961
C	-4.39177	0.00194	-0.24566
H	-0.89275	-1.28065	1.04327
H	-2.40434	-2.15598	1.31191
H	-4.00039	-1.28303	-1.95379
H	-4.22037	-2.15679	-0.43191

H	-2.38096	-0.00199	2.57264
C	-4.10454	0.00077	1.26299
H	-5.47318	0.00335	-0.41315
H	-4.55593	-0.88223	1.72865
H	-4.55381	0.88434	1.72961
Cl	1.28256	-0.00112	-0.64777
C	2.94381	0.00002	-0.00840
Cl	3.18743	1.44949	0.98816
Cl	4.10668	-0.00022	-1.34805
Cl	3.18880	-1.44820	0.98967

Cl abstraction from CCl_4 – transition state in dichloromethane

30

-2268.836283

C	-1.34484	1.06143	0.36895
H	-1.12708	2.07691	0.69060
C	-1.96806	0.15035	1.38463
C	-1.89261	0.89363	-1.01778
H	-1.49563	0.27987	2.36136
C	-1.88831	-1.31663	0.92899
C	-3.46247	0.57336	1.46451
H	-1.36567	1.54247	-1.72134
C	-1.81621	-0.57624	-1.46737
C	-3.38721	1.31471	-0.93036
H	-2.40032	-1.94852	1.66187
H	-0.84412	-1.63818	0.88741
C	-2.54445	-1.46880	-0.45134
H	-3.54024	1.61056	1.80511
H	-3.96817	-0.05780	2.20282
C	-4.11315	0.41589	0.08191
H	-0.77245	-0.88812	-1.56227
H	-2.28164	-0.67155	-2.45372
H	-3.46321	2.36394	-0.62825
H	-3.83906	1.22165	-1.92348
H	-2.47712	-2.51259	-0.77127
C	-4.01830	-1.04944	-0.36764
H	-5.16329	0.71670	0.14163
H	-4.49743	-1.16969	-1.34530
H	-4.55218	-1.69095	0.34169
Cl	0.87696	0.48822	0.22435
C	2.79197	-0.00719	-0.00477
Cl	3.69291	1.45240	-0.39512
Cl	2.88347	-1.17837	-1.31744
Cl	3.34957	-0.71407	1.51027

Cl abstraction from CCl_4 – products 47 and CCl_3 radical in dichloromethane

30

-2268.8829758

C	-1.58604	-1.46703	0.00019
H	-2.21505	-2.35788	0.00028
C	-1.83404	-0.63474	-1.25885
C	-1.83402	-0.63445	1.25903
H	-1.61769	-1.24319	-2.14078
C	-0.96150	0.62779	-1.25309
C	-3.32163	-0.23217	-1.25070
H	-1.61771	-1.24271	2.14110
C	-0.96148	0.62805	1.25306
C	-3.32161	-0.23182	1.25079
H	-1.16916	1.21111	-2.15594
H	0.09560	0.34834	-1.27951
C	-1.26408	1.46426	-0.00013
H	-3.95438	-1.12586	-1.26913
H	-3.54069	0.34311	-2.15577
C	-3.62563	0.60597	-0.00007
H	0.09574	0.34882	1.27958
H	-1.16921	1.21159	2.15575
H	-3.95437	-1.12548	1.26961
H	-3.54060	0.34378	2.15568
H	-0.63892	2.36138	-0.00026
C	-2.74716	1.86611	-0.00020
H	-4.68150	0.89140	-0.00010
H	-2.97002	2.47461	0.88295
H	-2.97000	2.47439	-0.88351
Cl	0.11380	-2.14257	0.00013
C	2.54831	0.26743	-0.00006
Cl	3.07425	-0.46121	1.46149
Cl	2.40267	1.97875	-0.00008
Cl	3.07406	-0.46143	-1.46152

BrO^{t}Bu Homolysis - BrO^{t}Bu Optimised Geometry in dichloromethane

15

-246.3067183

C	-1.27184	0.00002	0.03466
C	-1.29606	1.26315	0.88399
H	-0.47035	1.27354	1.59818
H	-1.22662	2.14805	0.24799
H	-2.23046	1.30891	1.44693
C	-1.29667	-1.26185	0.88579
H	-2.23005	-1.30539	1.45059
H	-1.22963	-2.14780	0.25101
H	-0.46966	-1.27236	1.59849
C	-2.41975	-0.00035	-0.97642
H	-2.37324	0.88876	-1.60696
H	-2.37279	-0.88956	-1.60682
H	-3.36878	-0.00058	-0.43652
O	-0.13765	-0.00115	-0.86009
Br	1.56511	-0.00001	-0.02944

BrO^{t}Bu Homolysis - Br Radical Optimised Geometry in dichloromethane

1

-13.2872674

Br	0.00000	0.00000	0.00000

BrO^{t}Bu Homolysis - O^{t}Bu Radical Optimised Geometry

in dichloromethane

14

-232.9600216

C	0.00013	-0.02598	0.07633
C	-1.26562	-0.79290	-0.31355
H	-1.29097	-1.75801	0.19702
H	-2.15303	-0.22279	-0.03306
H	-1.28435	-0.97201	-1.39063
C	1.27237	-0.78234	-0.31324
H	1.29295	-0.96129	-1.39032
H	2.15486	-0.20482	-0.03242
H	1.30554	-1.74719	0.19736
C	-0.00569	1.38003	-0.57554
H	-0.89906	1.93341	-0.28471
H	0.88274	1.94106	-0.28425
H	-0.00486	1.24269	-1.65861
O	-0.00136	0.25952	1.42945
